# Removal of Heavy Metals from Wastewaters and Other Aqueous Streams by Pressure-Driven Membrane Technologies: An Outlook on Reverse Osmosis, Nanofiltration, Ultrafiltration and Microfiltration Potential from a Bibliometric Analysis

**DOI:** 10.3390/membranes14080180

**Published:** 2024-08-22

**Authors:** Katherinne Castro, Ricardo Abejón

**Affiliations:** Departamento de Ingeniería Química y Bioprocesos, Universidad de Santiago de Chile (USACH), Av. Libertador Bernardo O’Higgins 3363, Estación Central, Santiago 9170019, Chile

**Keywords:** heavy metals, pollution, wastewater treatment, microfiltration, ultrafiltration, nanofiltration, reverse osmosis, bibliometric analysis, research trends

## Abstract

A bibliometric study to analyze the scientific documents released until 2024 in the database Scopus related to the use of pressure-driven membrane technologies (microfiltration, ultrafiltration, nanofiltration and reverse osmosis) for heavy metal removal was conducted. The work aimed to assess the primary quantitative attributes of the research in this field during the specified period. A total of 2205 documents were identified, and the corresponding analysis indicated an exponential growth in the number of publications over time. The contribution of the three most productive countries (China, India and USA) accounts for more than 47.1% of the total number of publications, with Chinese institutions appearing as the most productive ones. Environmental Science was the most frequent knowledge category (51.9% contribution), followed by Chemistry and Chemical Engineering. The relative frequency of the keywords and a complete bibliometric network analysis allowed the conclusion that the low-pressure technologies (microfiltration and ultrafiltration) have been more deeply investigated than the high-pressure technologies (nanofiltration and reverse osmosis). Although porous low-pressure membranes are not adequate for the removal of dissolved heavy metals in ionic forms, the incorporation of embedded adsorbents within the membrane structure and the use of auxiliary chemicals to form metallic complexes or micelles that can be retained by this type of membrane are promising approaches. High-pressure membranes can achieve rejection percentages above 90% (99% in the case of reverse osmosis), but they imply lower permeate productivity and higher costs due to the required pressure gradients.

## 1. Introduction

Heavy metals are toxic at low concentrations and dangerous because they tend to bioaccumulate, that is, to increase their concentration in a biological organism over time, accumulating and putting life at risk [1]. The main polluting sources are industrial and mining activities since they can release toxic metals such as lead, mercury, cadmium, arsenic and chromium into the environment, which, as already mentioned, are very harmful to human health and for most forms of life [2,3]. Another polluting source is caused by the combustion of fuels, generating material suspended in the atmosphere, which ends up being deposited in bodies of water or dragged from other surfaces by runoff into bodies of water [4,5]. Finally, untreated or inadequately treated wastewaters from mines and industrial activities can reach natural water bodies [6,7,8], while different industrial and other solid wastes can contaminate groundwater and surface waters when they are not effectively managed [9,10,11]. Agricultural activities can also lead to the pollution of soil and water sources with heavy metals through the use of contaminated fertilizers, pesticides and irrigation water [12,13]. This contamination can accumulate over time, posing risks to food safety, ecosystems and human health.

**Figure 1:**
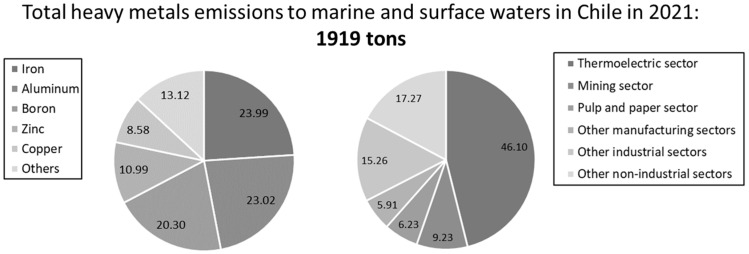
Distribution percentages of heavy metals emitted to marine and surface waters in Chile during the year 2021 (**left side**) and percentage contribution of the different sectors (**right side**). Data from Ministerio de Medio Ambiente de Chile, 2023 [14].

In the Chilean case, the industrial activity that contributes the most to heavy metal emissions into marine and surface waters is the thermoelectric sector, accounting for 46.1% of the total, representing 888.6 tons in 2021 [14]. Iron, aluminum and boron must be highlighted as the most frequent heavy metals in these Chilean emissions, with contributions above 20% for three pollutants (Figure 1). Regarding the emissions to groundwaters, they are concentrated in the Metropolitan Region, primarily associated with agricultural activity, which accounts for 97.7% due to 13.1 tons in 2021, with iron being the most frequent heavy metal (higher than 98.7%), followed by manganese.

Since water pollution with heavy metals is a great concern, numerous technological treatment methods have been developed to prevent the increase in heavy metal contamination. There are several physical, chemical and biological treatment options available for the removal of heavy metals from water, including chemical precipitation, ion exchange, electrochemical technologies, adsorption, biochemical technologies and membrane technologies [15,16,17,18,19,20,21]. Membrane technologies are basically based on the passage of liquid flow that permeates through a semipermeable membrane, producing the separation of the feed streams into two effluents: the permeate and the retentate streams. The membrane that separates these two streams acts as a barrier with very specific characteristics: the membrane must be permselective, allowing the passage of certain substances while others are retained. These technologies are useful for water treatment, due to the reduced energy consumption when compared to technologies that imply phase changes (like distillation), the avoided solid waste production linked to other technologies (like precipitation), the high efficiency in the retention of pollutants and the ease of integration with different traditional processes [22]. For the transport of components through the membrane to occur, the action of a driving force on the feed stream is necessary. This driving force can be of different types depending on the nature of the membrane technology, such as chemical potential, electrical potential and hydrostatic pressure [23]. Specifically, this document will focus on the membrane technologies driven by a hydrostatic pressure gradient: microfiltration, ultrafiltration, nanofiltration and reverse osmosis [24].

The fundamentals of microfiltration involve the use of a microporous membrane (typical pore size range between 0.1 and 10 µm) to separate suspended particles and other impurities from a liquid stream. This technology relies on the physical size of the particles being filtered, with membranes typically designed to capture particles larger than their pore size ratings. Microfiltration is used across a wide range of industries, including biopharmaceutical production, food and beverage processing and wastewater treatment. It is often considered a low-cost option as it does not require additional chemicals or high pressure levels. Microfiltration usually serves as a pretreatment for other stages based on membrane technologies [25]. Therefore, the proper selection and maintenance of the membranes are critical to ensure optimal performance of the complete separation process. Nevertheless, microfiltration enables high efficacy in removing heavy metals associated with suspended solids or colloids, while leaving behind other soluble species [26].

Ultrafiltration is a process used to separate molecules of high molecular weight from solutions with low levels of solute concentration. This process involves the use of a semipermeable membrane with pores of varying sizes (from 0.01 to 0.1 µm) that allow smaller molecules to pass through while retaining larger ones. The concept of molecular weight cut-off is critical in ultrafiltration as it determines the maximum size of molecules that can pass through the membrane. This cut-off is defined as the minimum molecular weight of a solute that is 90% retained by the membrane [27]. The molecular weight cut-off value of a membrane is determined by factors such as the pore size, shape, and distribution. By controlling the molecular weight cut-off of the membrane, ultrafiltration can be used for a variety of applications, including the removal of heavy metals when other techniques are employed to include the metals in molecules with high molecular weight, like complexes or micelles [28,29,30].

Nanofiltration is a membrane technology that operates on the nanometer scale (pore sizes below 0.01 µm), acting as a barrier to selectively separate solutes based on size and charge. In fact, the role of size and charge is critical in nanofiltration, as it determines the types of species that can pass through the membrane. The size of the species to be retained is determined by the pore size of the membrane, which can range from 1 to 10 nanometers. However, the charge of the molecules also plays a significant role in their ability to pass through the membrane. Charged species with functional groups are more likely to be retained by the membrane due to their interaction with the charged groups on the membrane surface. Actually, the higher the effective charge of the species, the lower the possibility of permeating across the membrane [31,32]. The ability of nanofiltration to remove heavy metals, such as lead, cadmium and copper, from contaminated waters has been tested in numerous studies, showing high removal efficiencies ranging from 80 to 99% [33].

Reverse osmosis membranes are characterized by requiring high pressures to carry out the separation, working within a range between 15 and 75 bar [34]. This occurs because the pores of these membranes are below the nanometer scale, so reverse osmosis membranes can be considered dense (at least their effective active layer). In this case, the species (water as solvent and ions as solutes) are subject to complex interactions with the membrane materials, showing phenomena like steric inclusion, the Donnan effect, dielectric exclusion, diffusion, advection and electromigration [35]. These mechanisms result in very high retention of heavy metals, with rejection percentages above 99% even for monovalent ions [36]. Reverse osmosis technology has been extensively used in large-scale industrial applications such as desalination plants, as well as smaller-scale specific systems. Despite being energy-intensive compared to other membrane technologies (which implies higher costs), reverse osmosis remains one of the most efficient options for achieving a high removal of dissolved metallic ions [37,38].

Numerous scientific records concerning water pollution and wastewater treatment are archived in databases. To effectively handle this extensive amount of bibliographic information, the utilization of specific tools becomes essential. Bibliometric resources, as valuable aids, prove indispensable in the systematic management of such a vast amount of scientific literature. Pritchard (1969) defined bibliometrics as the application of mathematical and statistical techniques to analyze various knowledge communication formats, including books. Within the framework of library and information sciences, this research methodology has been successfully employed to quantitatively depict distribution patterns of documents based on specified categories such as year, knowledge category, country, institution, author or source. This approach provides a substantial advantage in systematically examining, organizing and analyzing the large volume of scientific information. This way, pertinent information and knowledge about the state of the art within distinct disciplines become attainable, which helps researchers to identify innovative research patterns. Regarding the specific case of water pollution and wastewater treatment options, several bibliometric studies covering this topic have been recently published [39,40,41,42,43,44,45,46,47], but none was focused on the potential of membranes assisted by pressure to remove heavy metals.

The objective of this study was to conduct a bibliometric analysis of the scientific literature available in Scopus regarding the application of pressure-driven membrane technologies for removing heavy metals from wastewater. The documents identified through the bibliographic search were studied and evaluated based on various criteria, including annual production, the most prolific countries and institutions, main sources and languages. Through this analysis, the resultant quantitative attributes of the research were utilized to pinpoint significant trends and research voids in the field. This was achieved through the application of bibliometric network analysis to identify key topics within the research landscape.

## 2. Data Sources and Methodology

The search for scientific literature related to pressure-driven technologies (microfiltration, ultrafiltration, nanofiltration and reverse osmosis) for wastewater treatment to remove heavy metals was conducted using Scopus, a database containing abstracts and citations for over 84 million papers from more than 7000 international publishers [48]. Scopus is considered the most complete abstract and citation database of peer-reviewed literature, covering over 27,000 scientific journals and 249,000 books, including non-English titles with English abstracts. The database provides a comprehensive collection of peer-reviewed literature across STEM fields, with significant contributions from Europe, Latin America and Asia.

The search, completed in February 2024, used the search terms “*heavy metal*” AND (“*microfiltration*” OR “*ultrafiltration*” OR “*nanofiltration*” OR “*reverse osmosis*”) in the Article Title, Abstract and Keywords field. Quotations were used to limit the results to papers with both words in the exact order. The search was limited to 2023 to ensure a fair comparison with previous years (as 2024 was still not completed when the search was performed), resulting in 2205 documents.

This systematic bibliographic search provided insight into global research trends, identifying past research gaps, present hot topics and future research strategies. The study encompassed more than just a quantitative interpretation of publications according to annual production, most prolific countries and institutions, main journals or frequently used languages, since it involved identifying significant research themes by analyzing highly cited papers and frequently occurring keywords. In order to further examine the most important research topics and their evolution over time, a bibliometric network analysis using science mapping was conducted. This methodology allowed us to detect and visualize the conceptual subdomains, both particular and general, and provided insight into their thematic evolution [49]. An open-source software called SciMAT (v1.1.06) was employed because of its effective deduplication process and useful visualization tools based on strategic diagrams and thematic areas. The analysis was conducted in four phases taking into account three different subperiods (1972–2010, 2011–2020 and 2021–2023, with 560, 965 and 680 documents, respectively).

Firstly, research themes were identified within restricted networks in terms of size by limiting the frequency of keywords, with a minimum frequency of 35. This way, all the keywords with frequency values below this threshold were not considered. The simple center algorithm for clustering was selected with an edge value equal to 5. To normalize the data, the equivalence index for the co-word network of the thematic evolution structure was preferred. Secondly, the previously identified themes were plotted on a 2D diagram composed of four quadrants, where the y- and x-axes denote the normalized density and the centrality of a theme, respectively [50]. Thirdly, the outcomes were systematically structured into thematic network formations as clusters, yielding the corresponding thematic evolution structure. These thematic network structures delineate the co-occurrence patterns among research themes, accentuating the quantity of relationships and internal cohesion among them. The thematic evolution structure offers a holistic perspective on how these themes maintain a conceptual connection across defined subperiods. The size of the clusters is proportionate to the number of core documents, with links indicating co-occurrence among them. Solid lines denote clusters sharing the main theme, while dashed lines signify the sharing of other elements among clusters (Furstenau et al., 2021 [51]). The thickness of the lines corresponds to the inclusion index, reflecting elements in common among the themes. Lastly, the scientific contribution of the most relevant research topics was quantitatively assessed through metrics such as the centrality and density values.

## 3. Results and Discussion

### 3.1. Bibliometric Analysis of Research Trends in Pressure-Driven Membrane Technologies for Heavy Metal Removal (1972–2023)

#### 3.1.1. Publication Year, Document Type and Language of Publications

**Figure 2:**
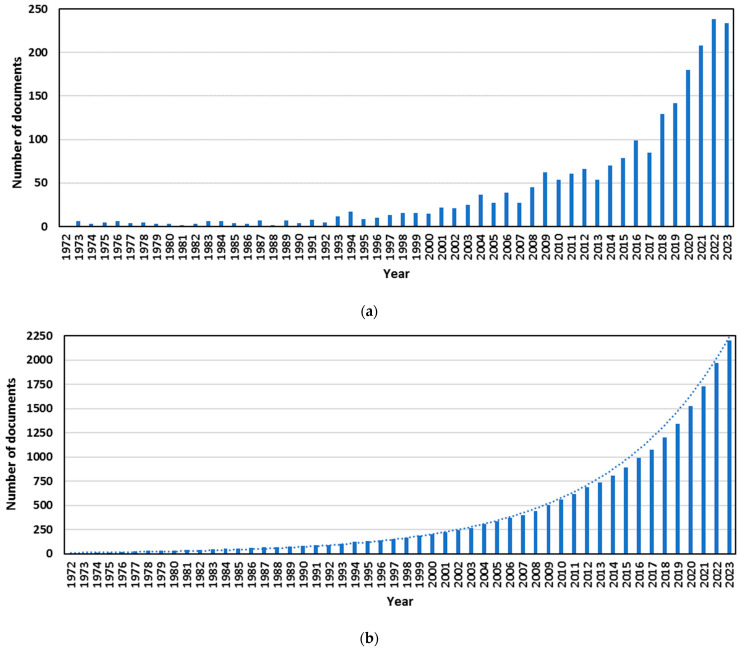
Annual (**a**) and accumulated (**b**) publication output. The line in the graph of the accumulated publication output represents the exponential fitting.

Figure 2 illustrates the graphical representation of the progression of annual scientific production identified in the Scopus database and the cumulative total of documents over time. Since the publication of the pioneering works published in 1972 and 1973, where reverse osmosis started to be applied for the removal of heavy metals from wastewater [52,53,54], until 1993, the number of documents published each year did not exceed 10. In fact, the 1990s marked a turning point in global environmental concern following the publication of the Brundtland Report in 1987 and the United Nations Conference on Environment and Development, commonly known as the ‘Earth Summit’, in 1992 [55,56,57,58]. The issue of adequate management of water resources became a priority during this period [59,60], and consequently, the number of published papers on the treatment of wastewater polluted with heavy metals using selected pressure-driven membrane technologies has steadily increased with an accelerated growth rate since the 1990s. Upon analyzing the accumulated number of publications related to pressure-driven membrane technologies for the removal of heavy metals from polluted wastewater, it was observed that the corresponding evolution can be fitted by an exponential growth equation. The obtained equation, y = 9.960 × e^0.1042·x^, where y represents the number of accumulated documents and x the year (starting in 1 for the year 1972), was applied to the data, resulting in a good fitting with an R^2^ value of 0.945. This finding confirmed that reverse osmosis, nanofiltration, ultrafiltration and microfiltration were deemed hot topics in this research area and had been experiencing continuous growth with an accelerated growth rate since the 1990s. A closer examination of the last two decades of the analyzed time range revealed that the number of documents published from 2013 to 2021 was lower than the projection based on the exponential growth model. This observation could suggest a deceleration in the rate of scientific production in this field, which could be justified by recent advancements in alternative membrane technologies not based on applied pressure, such as electrodialysis, membrane distillation and forward osmosis, that have experienced significant growth in the past few decades [61]. This trend may indicate a shift in research focus from traditional pressure-driven membrane technologies to more innovative approaches, but the production rates in 2022 and 2023 were closer to the predictions by the exponential model, so pressure-driven membrane technologies are still a relevant option for heavy metal removal from wastewater.

The different types of documents were evaluated, and 13 categories were identified. Article (1613 documents) was the most frequent document type, contributing 75.15% of the total production, followed by Review (243) and Conference Paper (166), which contributed 11.02% and 7.53%, respectively. Book Chapter (147) contributed 6.67% of the total production, and the other less significant categories jointly contributed 1.63%: Conference Review (17), Book (5), Note (4), Retracted (3), Short Survey (2), Editorial (2), Data Paper (1), Report (1) and Letter (1). The results indicate that the supremacy of articles over other types of publications is consistent with previous studies conducted in this field regarding water pollutants and treatment technologies [39,40,62]. However, the analysis of these previously published works revealed that second position of the ranking covering the document types is not as clear as the leading position of Article. On the one hand, the importance of contributions in congresses is significant in several engineering fields, including chemical, civil and environmental [63,64]. On the other hand, some examples with higher relative importance of Review over Congress Paper can be found [65,66,67]. In this work, the contribution of Review was higher than that of Conference Paper. The significant amount of published documents found in the search (more than 2200) forms a great corpus to be reviewed and summarized by the corresponding reviews, but the importance of the contributions in congresses was worth mentioning, confirming that the application of pressure-driven membrane technologies for water removal is still a hot topic widely considered in international scientific congresses and conferences.

The analysis of language usage revealed that English predominated as the most frequently employed language within the scientific community for publishing documents on the investigated topic, constituting 94.39% of the total documents. Chinese and German followed as the second and third most utilized languages, representing 2.76% and 0.50% of the overall number of papers, respectively. Other identified languages were considered anecdotal, as they did not exceed 10 documents (Table 1). These results demonstrate that English has undoubtedly become the global lingua franca in the scientific research literature, particularly in the engineering and natural sciences, where more than 90% of the total publications are written in English [68,69].

#### 3.1.2. Publication Distribution of Countries and Institutions

The list of the top 14 countries based on their total number of published papers is shown in Table 2, taking into account that only those countries have published at least 60 documents. However, it should be noted that the affiliation of a country is not an exclusive category, as a document can be the result of the collaborative efforts of researchers from multiple countries, thus leading to the possibility of a document being linked to more than one country simultaneously. Classically, a select group of leading nations, with the USA and China prominently positioned as global research leaders, tends to dominate international scientific production. However, in this instance, although China emerged as the foremost contributor with 471 documents, accounting for 21.36% of the total, India claimed the second position with 356 documents and a 16.15% contribution, surpassing the scientific output of the United States, which produced 213 documents (9.66% contribution). The cumulative contribution of these three nations constitutes above 47% of the overall document count. Besides China and India, other Asian countries, including Iran, Malaysia, South Korea and Saudi Arabia, also secured positions in the ranking. There could be three main reasons to justify this contribution. Firstly, countries like India, Iran and Saudi Arabia are highly affected by hydric stress [61]; therefore, treating wastewater polluted with heavy metals could be a viable option for water recovery. Secondly, certain Asian countries are dealing with water bodies severely polluted with heavy metals [70,71,72,73,74], demanding the development of effective treatment options. Finally, South Korea can be deemed a significant player in the global scientific community [75,76,77,78], and its collaboration and that from other relevant countries like Japan and Singapore with other research institutions in Asia are robust [79,80,81]. Regarding European contributions, it is noteworthy that the principal research powers of the continent appeared in the ranking: United Kingdom, Spain, Germany, Italy and France published 76, 72, 70, 66 and 61 documents, respectively.

Table 3 contains a compilation of the top 11 institutions, the only ones with at least 20 documents published. Two Chinese institutions, the Ministry of Education and the Chinese Academy of Sciences, shared the first position of the ranking, both with 51 documents. This amount only represented 2.31% of the total number of papers published, which revealed the high diversity of different institutions that have applied research efforts to the topic analyzed in this work. The podium was completed with the University Teknologi Malaysia, with 44 documents. In fact, a more detailed outlook on the list of institutions from Table 3 revealed that all of them were located in Asian countries. Surprisingly, no institutions from the USA or European countries appeared in the list even though the USA was the third most productive country and five European countries were identified among the most productive ones. Hence, the production from the United States and the most important European countries (United Kingdom, Spain, Germany, Italy and France) was widely distributed among different institutions, as none of them published at least 12 documents.

#### 3.1.3. Distribution of Output in Subject Categories and Journals

Table 4 presents the distribution of subject categories, which identifies the top ten subject categories, the only ones with at least 100 documents. As some papers may belong to more than one category, the sum of the number of documents exceeds the total number of identified papers. The ranking of the top ten categories reveals that *Environmental Science* is the most prevalent subject, contributing 51.93% (1145 documents), followed by *Chemistry* (36.19% and 798 documents) and *Chemical Engineering* (34.92% and 770 documents). In addition, two other related subjects, *Engineering* and *Materials Science*, rank fourth and fifth, with 579 and 501 documents, respectively. These discoveries underscore three distinct approaches to the examined topic. Firstly, an emphasis is placed on the environmental perspective, particularly addressing the presence of heavy metal pollutants in water bodies. Secondly, engineering works concentrate on the quest for technically effective solutions to treat wastewater contaminated with heavy metals, with a particular focus on traditional membrane technologies within the field of chemical engineering. Thirdly, the contributions of chemists and materials scientists are pivotal, as they actively engage in the development of novel materials and membranes, advancing the understanding of associated mechanisms and playing a vital role in the global research landscape. Taken together, these results provide compelling evidence of interdisciplinary research efforts aimed at implementing technical solutions based on innovative aspects of traditional pressure-driven membrane technologies. This combined strategy has the potential to alleviate the challenges associated with heavy metal pollution in water.

The number of documents published in the most frequent journals is compiled in Table 5. As indicators of the journal relevance, the corresponding SCImago Journal Ranking (SJR) index, Impact Factor (IF) and the Journal Citation Indicator (JCI) of the top 14 journals, the only ones that published at least 25 articles, were included. The Journal Citation Indicator (JCI) is the average Category Normalized Citation Impact (CNCI) of citable items (articles and reviews) published by a journal over a recent three-year period. The average JCI in a category is 1. Journals with a JCI of 1.5 have 50% more citation impact than the average in that category. This index can give a much clearer idea about the absolute relevance of a journal, in opposition to the SJR and IF indices, which are more useful within a relative framework that allows the direct comparison of different journals. Only the two leading journals *Journal of Membrane Science* and *Desalination* contributed more than 4% to the total scientific production (109 and 99 documents, implying contributions of 4.94% and 4.49%, respectively). On the one hand, *Journal of Membrane Science* provides a focal point for academic and industrial chemists, chemical engineers, materials scientists and membranologists working on membrane systems, since it publishes original research and reviews on membrane transport, membrane formation and structure, fouling, module and process design, and applications. On the other hand, *Desalination* is distinctly centered on water desalination, encompassing the application of desalination techniques to seawater, groundwater and wastewater through thermal, membrane, sorption and hybrid processes. Both journals have high quality parameters; for instance, the IF values were close to 10 (9.5 and 9.9 for *Journal of Membrane Science* and *Desalination*, respectively). Indeed, three other journals included in the ranking presented IF values above 10: *Journal of Hazardous Materials* in the sixth position (IF equal to 13.6), *Chemical Engineering Journal* in the seventh position (IF equal to 15.1) and *Water Research* in the ninth position (IF equal to 12.8). A more general journal completed the podium with 63 documents: *Chemosphere*. This journal is dedicated to the presence of chemicals in the environment and covers investigations related to all aspects of the identification, quantification, behavior, fate, toxicology, treatment and remediation of chemicals in the biosphere, hydrosphere, lithosphere and atmosphere. Only one other journal published at least 70 papers (just 70 for 3.17% contribution): *Desalination and Water Treatment*, which is dedicated to research and application of desalination technology, environment and energy considerations, integrated water management, water reuse, wastewater and related topics. A concise review of the topics covered in these four leading journals is adequate to reconfirm the three interdisciplinary perspectives integral to research in this field: environmental sciences, engineering and chemistry and materials sciences. Although the high relevance of most journals included in the ranking is confirmed by their IF and JCI values (with more JCI values above 1.45), papers on innovative aspects of pressure-assisted membrane technologies for heavy metal removal can be also found in journals with lower prominence, like *Water Science and Technology* (JCI = 0.43), *Separation Science and Technology* (JCI = 0.40) or *Desalination and Water Treatment* (JCI = 0.25).

#### 3.1.4. Most Frequently Cited Papers

Table 6 displays the top 10 articles arranged by the number of citations they have received. The citation range spans from 603 for the least cited article in the ranking to 6980 for the most widely referenced article in the top position. While a detailed analysis of the key topics, based on the most frequently selected author keywords, will be presented in the following subsection, examining the most cited publications offers an initial insight into the crucial aspects that have attracted the attention of researchers exploring metal removal from wastewater through pressure-driven membrane technologies.

The two most cited documents are generic reviews that cover the removal of heavy metals from wastewater [36,82]. In fact, four other papers in the list, the ones that occupied the third, fourth, sixth and eighth positions, were directly related to the treatment options for removing heavy metals from wastewater [83,84,85,86]. Another three documents in the list were reviews without a direct relationship to wastewater treatment by membrane technologies. The document in the fifth position reviewed the interactions of metal ions with chitosan-based sorbents [87], and the document in the seventh position covered surfactant-enhanced remediation of contaminated soil [88], while the document in the tenth position summarizes several areas of heavy metal remediation from a microalgal perspective [89]. In fact, the only document totally focused on membrane technologies for heavy metal removal from wastewater was the article in the ninth position of the ranking [90], which investigated the selective ion penetration and water purification properties of graphene oxide membranes. This advanced material revealed good properties for being employed in the fabrication of membranes, since sodium salts permeated through the synthesized membranes quickly, whereas heavy metal salts permeated much more slowly.

#### 3.1.5. Distribution Analysis of Author Keywords and Trending Topics of the Research

**Figure 3:**
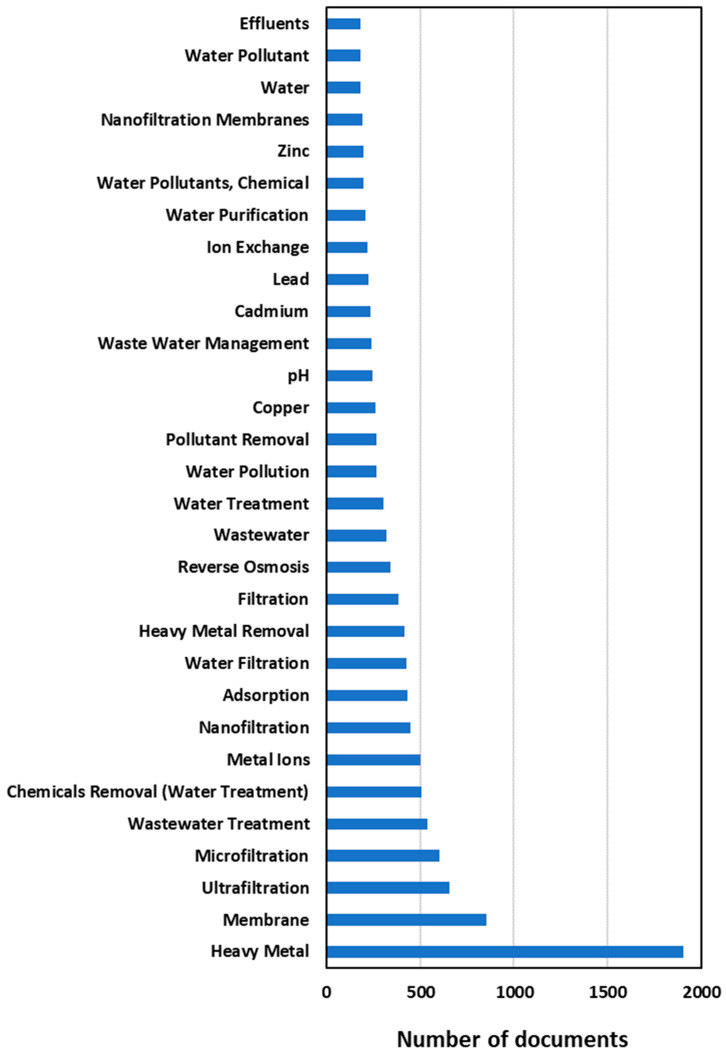
The top 30 most frequently used keywords (selected by at least 175 documents).

Figure 3 presents the list of the 32 most frequently used keywords, selected at least 175 times. Notably, “*heavy metal*” emerged as the most recurrent keyword, featuring in 1905 documents, which revealed that 86.4% of all identified papers utilized this keyword. The third position in the ranking was occupied by another term introduced in the Article Title, Abstract and Keywords field of the search-engine database: “*ultrafiltration*” appeared 657 times (29.8% of the documents), followed by “*microfiltration*”, another technology used in the search string, which was selected 604 times (27.3% of the documents). Upon further examination, the analysis of the two other additional technologies unveiled 450 occurrences for “*nanofiltration*” (20.4% of the documents) and 340 for “*reverse osmosis*” (15.4% of the documents). Interestingly, the prevalence of low-pressure membrane technologies (microfiltration and ultrafiltration) over high-pressure technologies (nanofiltration and reverse osmosis) was evident in terms of the relative frequency of keywords. Microfiltration and ultrafiltration are based on porous membranes, with pore sizes above 0.01 µm (smallest pore diameters in ultrafiltration membranes), which are not adequate for the removal of heavy metals from wastewater, especially in the case of dissolved metallic ions. Nevertheless, these technologies can play relevant roles in the removal of heavy metals from wastewater.

On the one hand, microfiltration modules are often used as a pretreatment for nanofiltration or reverse osmosis, especially for streams with high fouling potential where the use of cartridge filters or other mechanical systems is not enough to reduce the silt density index to the required values [91]. Therefore, microfiltration can be coupled with a cartridge or other filtration method, such as sand filtration, to provide adequate characteristics in the feed stream of the nanofiltration and reverse osmosis stages [92]. Although this low-pressure technology is not capable of removing the dissolved salts contained in wastewater, it can remove suspended solids and avoid subsequent damage to nanofiltration or reverse osmosis modules [93]. In addition to particulates, organic compounds, microorganisms or trace amounts of free chlorine in the feed stream should be removed before it is fed into the membrane system as these are harmful to the nanofiltration and reverse osmosis membranes [94]. Microfiltration is more effective than sand filtration or other mechanical pretreatments in removing organic macromolecules, and COD reductions above 65% can be achieved when treating plating wastewater [95]. This way, microfiltration can significantly reduce fouling in nanofiltration and reverse osmosis stages and ensure the longevity and proper operation of these membrane modules [96]. For example, the extent of the fouling across the surface of a cellulose acetate reverse osmosis membrane without previous microfiltration covered more than 70% of the membrane surface, while only about 25% of the surface suffered fouling when microfiltration was installed as pretreatment [97]. This additional fouling decreased the reverse osmosis permeate flux from 0.716 to 0.481 L/m^2^·min, as observed when both cases were compared.

On the other hand, ultrafiltration membranes can remove metallic ions from wastewater when the process is assisted by added chemical reagents [33]. This way, two different approaches can be followed. The first approach is based on the addition of surfactants that produce micelles or complexing agents to increase the molecular weight and size of the metallic ions and therefore retain their passage through the porous layer via the size exclusion mechanism of ultrafiltration. The second approach is based on the incorporation of adsorbents with specific metal removal capacities into the ultrafiltration membrane matrix. The appearance of “*membrane*” in the second position of the ranking, with 853 times, must be highlighted, along with the presence of “*water filtration*” and “*filtration*” (428 and 386 times, respectively).

Concerning alternative treatment methods, both “*adsorption*” and “*ion exchange*” featured prominently among the most frequently used keywords, appearing 432 and 221 times, respectively. The presence of these keywords can be justified by the design of advanced treatment processes that combine several technologies [98,99,100,101], but also because adsorption and ion exchange mechanisms can be involved in the retention of heavy metals by membranes [102,103,104,105]. Keywords related to the objectives of treatments were frequent, with “*chemicals removal*”, “*heavy metal removal*” and “*pollutant removal*” being selected 509, 416 and 265 times, respectively. Additionally, terms like “*wastewater treatment*”, “*water treatment*”, “*waste water management*” and “*water purification*” were selected 538, 305, 238 and 210 times, respectively. Lastly, specific examples of heavy metals can be found in Figure 3: “*copper*” (259 times), “*cadmium*” (234 times), “*lead*” (226 times) and “*zinc*” (196 times). This list of metals can give a clear idea about the most investigated heavy metals, and most of them have been previously identified as a priority according to another bibliometric analysis focused on the use of innovative membrane technologies for metal removal from wastewater [61].

### 3.2. Bibliometric Network Analysis

**Figure 4:**
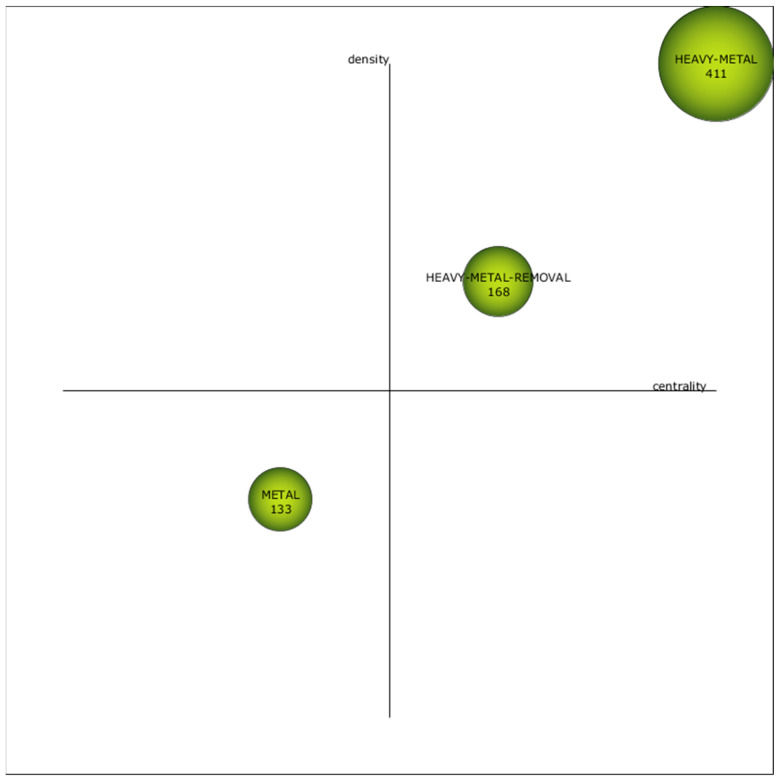
Strategic diagram of the first subperiod (1972–2010).

**Figure 5:**
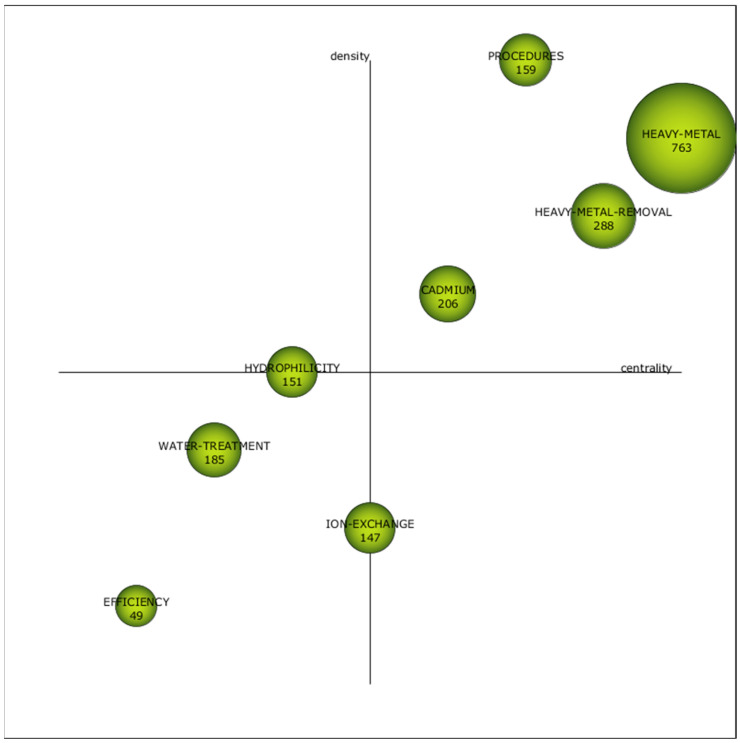
Strategic diagram of the second subperiod (2011–2017).

**Figure 6:**
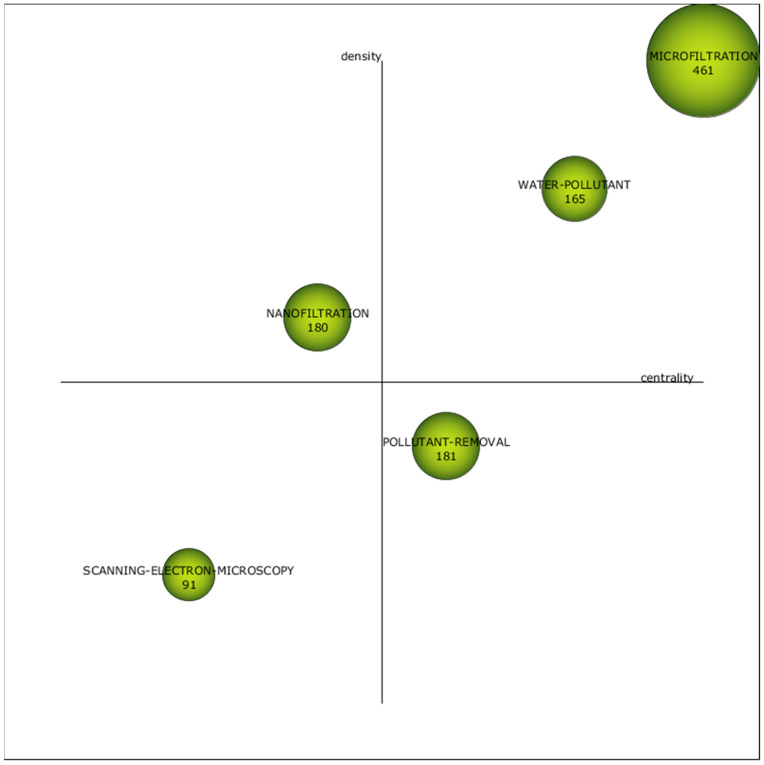
Strategic diagram of the third subperiod (2018–2021).

This section presents the key outcomes derived from the bibliometric network analysis of the scientific literature related to the removal of heavy metals from wastewater using pressure-driven membrane technologies. To begin, the strategic diagrams for each specified subperiod are illustrated: 1972–2010 (Figure 4), 2011–2020 (Figure 5) and 2021–2023 (Figure 6). According to their situation in these strategic diagrams, the themes can be classified into four different categories [106]:

(a) First quadrant (high centrality and high density): Motor themes. Themes that have emerged in the field of research showing significant advancements.

(b) Second quadrant (high centrality and low density): Basic and transversal themes. Themes with the potential to evolve into motor themes in the future, given their high centrality.

(c) Third quadrant (low centrality and low density): Emerging or declining themes. Themes that need further qualitative analysis to determine if they are emerging or declining.

(d) Fourth quadrant (low centrality and high density): Highly developed and isolated themes. Themes that have ceased to be in vogue due to the emergence of a novel concept or technology.

The strategic diagrams include 16 clusters in total; 8 of them are motor themes, 2 are basic and transversal, 4 are emerging or declining themes and 2 are highly developed and isolated themes. The size of the clusters is indicative of the number of documents (precise values provided within each cluster). Furthermore, Table 7 displays the absolute centrality and density values of the clusters.

Figure 7 displays the thematic evolution structure, elucidating the evolution of the research field across various subperiods examined in this study. Thus, the significance of each individual theme is depicted both by its cluster size and its relationships across the different subperiods. The internal structures of the different clusters are provided as Supplementary Materials (Figures S1–S16).

The cluster “Heavy Metal” (Figure S1) was the most relevant in Subperiod 1; it also appeared in Subperiod 2 with the highest number of publications (Figure S5), and it was renamed “Microfiltration” in Subperiod 3 (Figure S12), where it was the most important one once again. The other series of clusters with clear continuity through time was the one formed by “Heavy Metal Removal” in Subperiod 1 (Figure S2) and Subperiod 2 (Figure S6), transformed into “Water Pollutant” in Subperiod 3 (Figure S13).

The clusters “Heavy Metal” and “Microfiltration” were motor themes in the three subperiods, and they were the most important ones in terms of published documents and centrality values. The cluster “Heavy Metal” from Subperiod 1 included three pressure-assisted membrane technologies as nodes: “Microfiltration”, “Ultrafiltration” and “Reverse Osmosis”. The cluster “Heavy Metal” from Subperiod 2 also included three membrane technologies, but in this case, they were “Microfiltration”, “Ultrafiltration” and “Nanofiltration”. Finally, the cluster “Microfiltration” from Subperiod 3 only contained this technology. Further analysis revealed that there was not any node called “Nanofiltration” in the clusters from Subperiod 1; “Reverse Osmosis” appeared as node in the cluster ”Water Treatment” from Subperiod 2 (Figure S8), while “Nanofiltration” was the central node of its own cluster in Subperiod 3 (Figure S14), and both “Ultrafiltration” and “Reverse Osmosis” nodes formed part of the cluster “Pollutant Removal” in the last subperiod (Figure S15). Nanofiltration must be considered the most recent technology among the pressure-assisted membrane technologies, so its absence during the first subperiod can be justified. The cluster “Water Treatment” where the node “Reverse Osmosis” was located during Subperiod 2 fell in the third quadrant, so its relevance was clearly lower that the one exhibited by the cluster that contained the rest of technologies. This fact indicated a clear loss of interest among the research community regarding reverse osmosis during the 2010s. The last years represented in Subperiod 3 showed greater diversification, with the four technologies shared among three different clusters. As previously mentioned, the node “Microfiltration” was the central one in its own cluster, which was the most important motor theme, while the node “Nanofiltration” also was the central one in its cluster, but in this case, it was located in the fourth quadrant, being a highly developed theme but having low connection with the rest of clusters. Additionally, the cluster “Pollutant Removal” where the nodes “Ultrafiltration” and “Reverse Osmosis” were included in Subperiod 3 was categorized as a basic transversal theme located in the second quadrant. This cluster had significant continuity with the cluster “Water Treatment” from Subperiod 2 (which contained the node “Reverse Osmosis”). Therefore, while ultrafiltration seemed to get reduced its research relevance during the last years (moved from the first quadrant to the second one), reverse osmosis exhibited the opposite trend and moved from a cluster in the third quadrant to one in the second.

Once again, as in the case of the analysis of the keywords, the relevance of the low-pressure technologies over the high-pressure ones has been confirmed, as commented in previous publications [33,107]. Although reverse osmosis (the tightest membrane technology) systems have demonstrated almost complete removal of heavy metals, with rejection percentages above 98% [16], they require high applied pressure, and the associated energy costs make the selection of this technology less attractive, a fact that is enhanced when the elevated costs attributable to the regeneration of the membranes are also considered [108]. These reasons can justify the reduced research interest in reverse osmosis after 2010, when the alternative technology of nanofiltration was totally mature. Indeed, nanofiltration is an intermediate technique between the dense membranes of reverse osmosis and the porous membranes of ultrafiltration, which reduces significantly the energy consumption of the process when compared to reverse osmosis [109], but high effectiveness for heavy metal removal is maintained, with rejection percentages above 90% for most multivalent cations [110,111]. Although ultrafiltration membranes present pores that are too loose to retain metallic cations [112], the addition of auxiliary chemicals able to produce micelles or metallic complexes that can be retained by these porous membranes is a useful approach to take advantage of the high permeability of ultrafiltration while achieving adequate metal rejection values [30,113,114]. In addition, adsorptive ultrafiltration membranes can be synthesized by the incorporation of adsorbents with high adsorption capacity for heavy metal in the membrane’s internal structure or on the external surface [46,115,116,117]. This same strategy can be applied to the case of microfiltration membranes, which are characterized by increased pore size, giving micellar-enhanced microfiltration [118,119,120], complex-enhanced microfiltration [121,122,123,124,125,126] and adsorptive microfiltration [127,128,129,130] as a result. A detailed analysis of the bibliometric network revealed that the node “Micellar-Enhanced Ultrafiltration” was included in the cluster “Cadmium” from Subperiod 2 (Figure S7), which was a motor theme.

Regarding heavy metals, the four elements (copper, cadmium, lead and zinc) most frequently used as keywords, which were identified in the previous section, appeared in the clusters in all the subperiods. In Subperiod 1, the main cluster “Heavy metal” included the nodes “Copper”, “Cadmium” and “Zinc”, while the node “Lead” formed part of the cluster “Metal” (Figure S3) where two elements were included as nodes: “Nickel” and “Chromium”. The two most important clusters of Subperiod 2 incorporated the nodes related to the most frequent metals too. “Cadmium” was the central node of its own cluster, where “Copper” and “Zinc” were also included, while “Lead” formed part of the cluster “Heavy Metal Removal”. Once again, “Nickel” and “Chromium” appeared as nodes, in this case, in the structures of clusters “Heavy Metal Removal” and “Cadmium”, respectively. The latter cluster included an additional metal, since the node “Arsenic” can be found in its structure. In Subperiod 3, the four main metals were shared between two clusters: the nodes “Copper” and “Lead” were part of the cluster “Pollutant Removal”, and the nodes “Cadmium” and “Zinc” were included in the cluster “Scanning Electron Microscopy”. Both clusters included an additional metal: “Iron” was present in the cluster “Scanning Electron Microscopy”, while “Chromium” appeared in the cluster “Pollutant Removal”. This way, chromium was also present in clusters through the three subperiods.

This detailed analysis of the bibliometric network confirmed the importance of the research efforts applied to the treatment of wastewater where copper, cadmium, lead and zinc are present [131,132,133,134,135,136,137,138,139,140,141,142,143,144,145,146,147], but it also identified other four relevant elements that were not previously considered during the keyword analysis. Several examples of treatment of wastewater containing chromium by means of pressure-driven membrane technologies can be found [148,149,150,151], especially focused on the wastewater produced by the tannery sector. The vast industrial use of nickel has resulted in its extensive distribution in the environment, which could have a serious impact on human health. The removal of nickel from wastewater before disposal into the water bodies is required, and membrane technologies are effective for this purpose [152,153,154]. Iron is the most common heavy metal in the world and its removal from wastewater by the membrane technologies covered in this work has gained research interest [155,156,157,158]. Finally, although arsenic is sometimes not considered a heavy metal, this toxic element must be removed from water bodies in order to avoid environmental and health problems, and several examples of treatment by pressure-driven membrane technologies have been identified [159,160,161,162,163].

“Adsorption” and “Ion Exchange” were previously identified as relevant keywords during the corresponding analysis based on the data included in Figure 3. Actually, the node “Adsorption” was always included in clusters categorized as motor themes: “Heavy Metal Removal” in Subperiod 1, “Heavy Metal” in Subperiod 2 and “Microfiltration” in Subperiod 3. This fact gave a clear idea about the relevance of this technology in combination with pressure-driven membrane technologies. The use of adsorptive porous membranes has been mentioned, but the coupling of an adsorption pretreatment before the membrane technologies is a common approach, which has been proved as an effective solution to alleviate the fouling of the membranes [100,164,165,166]. In contrast, “Ion Exchange” only appeared in Subperiod 2 as the central node of its own cluster (Figure S10) and in Subperiod 3, forming part of the cluster “Pollutant Removal”; in both cases, it represented basic transversal themes in the second quadrant. Therefore, ion exchange was less relevant than adsorption, and this technology was not so frequently coupled to pressure-driven membranes, although some examples of ion exchange membranes for heavy metal removal have been identified [167,168,169,170].

Another relevant cluster not previously mentioned that acted as a motor theme was “Procedures” in Subperiod 2 (Figure S4), which exhibited the maximal density among all the clusters and had no continuity with the clusters from Subperiod 1. It presented several nodes related to water and wastewater processing, such as “Water Management”, “Water Purification”, “Waste Disposal (Fluid)”, “Water Pollutant” and “Water Pollutants (Chemical)”. The cluster “Hydrophilicity” (Figure S9) was a highly developed theme in Subperiod 2 and strongly linked to the cluster “Nanofiltration” from Subperiod 3. This property promotes higher water permeation through the membranes, and several studies have investigated different procedures for increasing the hydrophilicity of pressure-driven membranes [171,172,173,174]. These clusters contained other terms highly related to the most important properties of the membranes, such as the nodes “Surface Property”, “Contact Angle”, “Porosity” and “Pore Size”, which pointed to the great research efforts applied to the improvement of the membrane properties, especially since the 2010s. Since the nodes “Nanofiltration” and “Nanofiltration Membrane” also were included in these clusters, the surface properties of this technology can be highlighted as the most investigated during the last few years, paying special attention to the contributions from the field of nanotechnology, as highlighted by the presence of the nodes “Nanoparticle” and “Nanocomposite” in the clusters [175,176,177,178,179,180,181,182,183].

### 3.3. Review of Hot Topics and Perspectives

**Figure 8:**
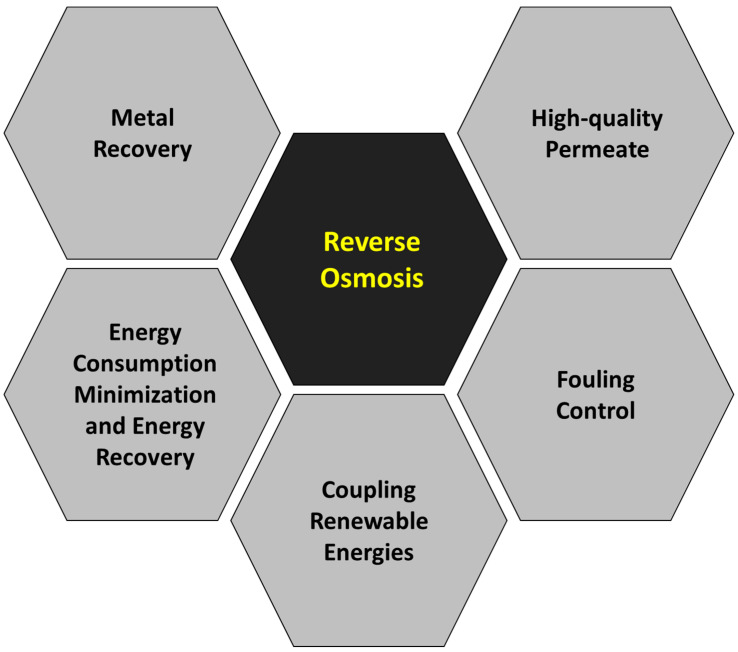
Main research topics related to reverse osmosis.

**Figure 9:**
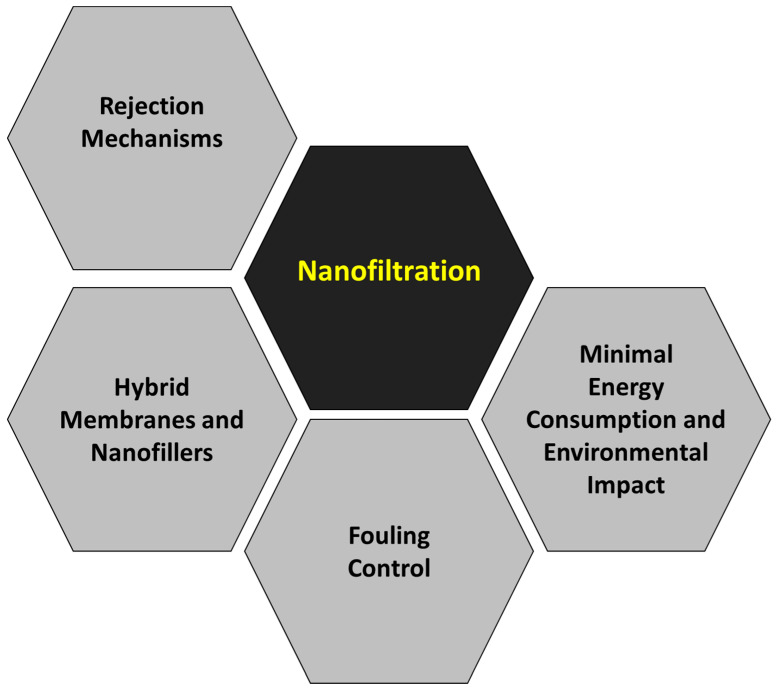
Main research topics related to nanofiltration.

**Figure 10:**
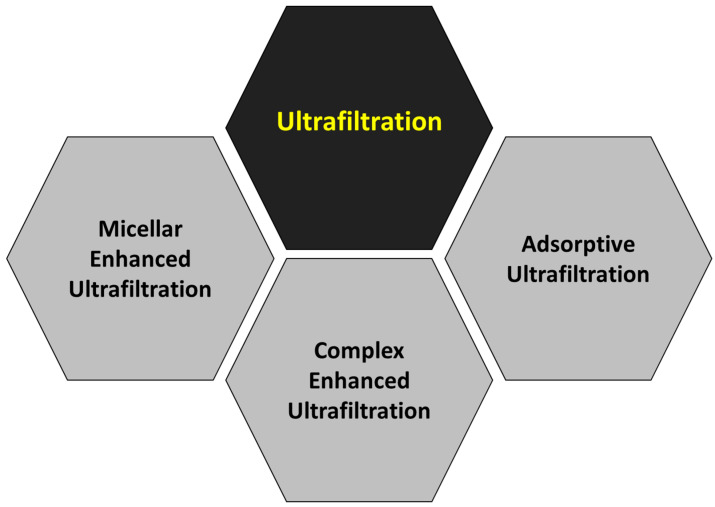
Main research topics related to ultrafiltration.

**Figure 11:**
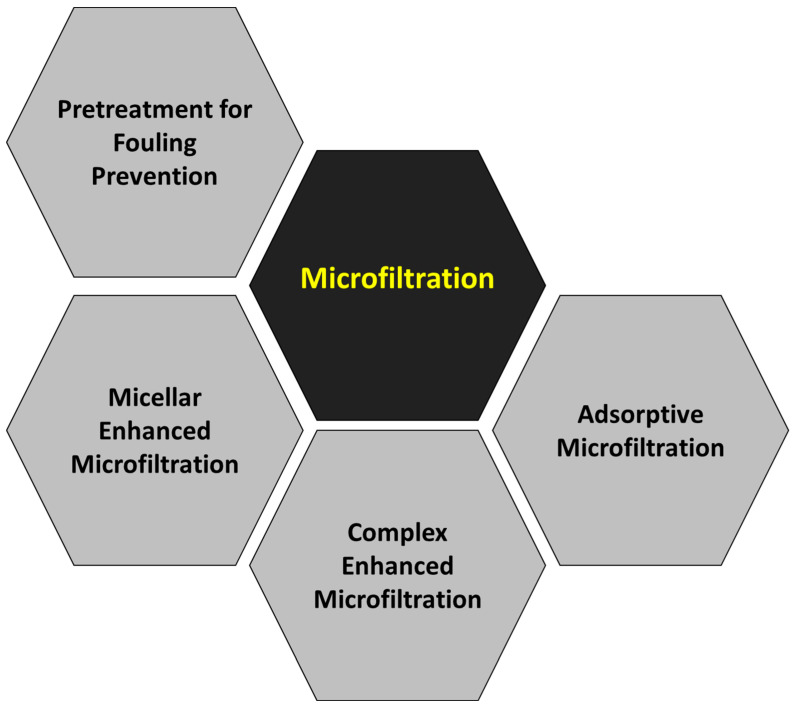
Main research topics related to microfiltration.

Although the main objective of this work is not an extensive and meticulous compilation of all the scientific papers published regarding the removal of heavy metals by pressure-driven membrane technologies, a brief summary has been prepared about the main topics studied in each membrane technology: reverse osmosis (Figure 8), nanofiltration (Figure 9), ultrafiltration (Figure 10) and microfiltration (Figure 11).

#### 3.3.1. Reverse Osmosis

Reverse osmosis membranes are the tightest ones among the pressure-driven technologies, and they can be considered dense without defined pores, so very high rejection percentages for almost all heavy metals have been reported [16,184]. Nevertheless, taking into account the low permeate productivity per unit of membrane area, the high operating pressure (above 15 bar) and the corresponding energy required to operate the reverse osmosis process, the removal of heavy metals by means of this technology is not the most preferable option in most cases, as other processes can offer similar performance with lower costs [185]. The energy consumption of reverse osmosis ranges between 3.5 and 5.5 kWh/m^3^, while nanofiltration requires from 1.5 to 2.5 kWh/m^3^. These figures can be significantly reduced when ultrafiltration is used (0.5–1.5 kWh/m^3^).

Nevertheless, some circumstances that justify the implementation of reverse osmosis membranes have been identified. For instance, the recovery of valuable heavy metals from wastewater can promote the use of reverse osmosis. In this framework, the implementation of reverse osmosis can provide environmental (decreased chemical consumption than alternative treatments) and economic (recovery of valuable products) benefits. Examples of systems designed to recover chromium [186] and zinc [141] from electroplating rinsing waters have been investigated. In the case of chromium, higher than 99.9% removal was achieved, and the retentate of the reverse osmosis process attained a chromium concentration equal to 8.4 g/L, which is adequate for direct recovery as demonstrated by Hull cell tests. In the case of zinc, the rejection rates were maintained at over 99% for different pH and initial concentration values, and a reaction with ferrite allowed the recovery of zinc ferrite free of chromium, the other metal present in the reverse osmosis retentate. Therefore, the assessment of optimal operation conditions and process configuration warrants the viability of the direct reuse of the recovered metals in electroplating tasks or the recovery of valuable solids and contributes to the circularity in such a relevant industrial sector.

In addition, another industrial sector that has taken advantage of processes based on reverse osmosis for the recovery of valuable heavy metals is the tannery sector. The toxicological impact of tannery wastewaters, which contain numerous potentially harmful pollutants, including chromium, is high, and they cannot be discharged to the environment without previous treatment. The selection of treatment options based on reverse osmosis allows the recovery of chromium [187]. The rejection of chromium can be around 99% at room temperature under neutral or slightly acidic conditions. This way, reverse osmosis permeate streams can fulfill the discharge limits imposed and allow a safe discharge of the treated wastewater, while the concentrate streams are enriched in chromium and must be considered as a valuable resource for reuse [151]. The high rejection selectivity to chromium in the presence of monovalent and divalent cations (with values higher than 8) exhibited by a membrane based on chitosan allowed the selective recovery of the chromium retained in the retentate of a previous reverse osmosis stage.

The production of high-quality permeate streams from treated wastewater can also favor the use of reverse osmosis membranes. The almost complete removal of heavy metals by reverse osmosis allows the attainment of very low concentrations in the permeate, even in the case of wastewaters with elevated high-strength or high initial metal contents, like mining wastewater [188]. The high-quality treated wastewater can thus be suitable for different reuse purposes, including the production of drinking water. Indeed, drinking water was obtained from acid mine drainage by reverse osmosis treatment after precipitation of metal hydroxides, gypsum and lime in previous pretreatment stages [189]. The coupling of different reverse osmosis stages in series allows the attainment of strict discharge limits even when high initial values are present in the raw wastewater. The feed stream to a wastewater treatment plant in Vietnam with a COD content above 30,000 mg/L and high metal concentrations (zinc and copper concentrations above 350 and 500 mg/L, respectively) was successfully treated by a train that contained two reverse osmosis coupled in series, allowing final zinc and copper concentrations below 3 and 2 mg/L, respectively [190].

The use of renewable energy sources alleviates the environmental impacts of the processes based on reverse osmosis [191,192,193]. Both wind and solar energies have been effectively coupled to feed reverse osmosis systems without the need for an electrical grid connection, and desalination plants under these conditions in Argentina can provide drinking water at competitive costs of around 0.6 USD/m^3^ [194]. The consideration of geothermal energy is also valid: the integration of solar and geothermal sources for water purification from Lake Ziway in Ethiopia demonstrated that the installation could be operated in both day and night cycles without problems [195]. Nevertheless, many efforts have been focused on the reduction in the specific energy consumption of reverse osmosis processes and the recovery of energy from these processes. Adequate pretreatments by gravitational filtration or low-pressure membranes can significantly reduce energy consumption [196,197], but the implementation of energy recovery devices [198,199] appears as a key aspect for minimal energy consumption in reverse osmosis treatments. The operation of reverse osmosis includes a highly pressurized retentate stream, and the recovery of its hydraulic energy implies a significant reduction in the total energy consumption. The most traditional energy recovery devices employed for reverse osmosis were centrifugal mechanisms like Pelton wheels, Francis turbines and Turbochargers [200,201,202,203], but isobaric chambers have been replacing these devices in more recent reverse osmosis installations [204]. These devices transfer the hydraulic energy directly from the retentate to the feed by means of direct contact with minimal mixing [205] and attain higher efficiency, with characteristic values above 95%, which cannot be attained by previous devices, since their maximal values are typically below 85% [206]. With increasing salinity of the stream to be treated, a higher applied pressure is required, and the specific energy savings that can be attained are higher [207].

The separation performance of the membrane is still the critical aspect to be improved in order to find an equilibrium between water permeability and solute rejection. Novel membrane materials and fabrication methods have greatly increased water permeability for reverse osmosis membranes while maintaining excellent rejection percentages for heavy metals as a result of the optimization of the composition, morphology and structure of the effective layers of these membranes. Innovative materials like fluoropolymers can be employed to produce reverse osmosis membranes with high chemical resistance and enhanced permeability. Fluorinated monomers such as 5-(trifluoromethyl)-1.3-phenylenediamine (TFPD), 3,5-bis(tri-fluoromethyl) benzoyl chloride (TFBC) and 2,2,2-trifluoroethylamine (TFEA) have been combined with polyamide, and the obtained membranes presented higher hydrophilicity and exceptional separation performance, maintaining salt rejection percentages above 99.6% [208]. Optimal interfacial polymerization using UV radiation improved the performance of polyamide reverse osmosis membranes [209]. By means of photo-Fries rearrangement of polyamide, the main membrane properties were modified, and increased permeability and rejection were achieved (15% and 1% increases, respectively). New approaches based on nanotechnology have been useful for these membrane modifications. The addition of graphene oxide nanosheets to a polyvinyl alcohol membrane enhanced its performance, showing less roughness, higher hydrophilicity and enhanced mechanical properties [210]. More complex nanofillers formulated with titanium dioxide, calcium alginate, chitosan and polyaspartic acid increased the water transport through the membrane while presenting excellent salt rejection (higher than 98%) and self-healing capacity to repair the surface damage suffered after contact with oxidant chemicals like chlorine [211]. In addition, the most adequate system designs and operating conditions have been discussed to improve water recovery and reduce energy consumption. The application of process and system engineering tools, mainly simulation and optimization, has allowed the identification of optimal design and operation conditions [212,213] and the design of more advanced configurations including recycle streams [214] or multistage systems [215], which can achieve a greater than 50% reduction in the energy consumption of the process.

Nevertheless, membrane fouling is still the most important challenge in reverse osmosis processes, and it reduces the performance and effective lifetime of this type of membrane [216,217,218]. Strategies for mitigating membrane fouling are a priority for maintaining adequate performance of the membrane as long as possible. In general, microfiltration and ultrafiltration pretreatment and physical and/or chemical cleaning appeared among the most effective strategies suggested by researchers to reduce fouling and ensure the long-term performance and proper operation of reverse osmosis systems [96]. On the one hand, the use of microfiltration and ultrafiltration membranes as a pretreatment can reduce the extent of the fouling across the surface of the reverse osmosis membranes and extend their effective lifetime for an additional 50% period [219,220,221]. On the other hand, fouling of reverse osmosis membranes is unavoidable in the long run even when proper pretreatment is applied, so membrane cleaning is required to restore the process performance. The cleaning strategies depend on the nature of fouling and can be chemical, physical or both in some cases. Reviews that present the most adequate physical and chemical cleaning methods have been published [222,223,224].

The design of specific robust antifouling reverse osmosis membranes has gained attention. Membrane properties, such as charge, morphology, chemical structure or hydrophilicity, greatly affect a membrane’s tendency to suffer fouling [225]. Hence, the development of novel materials, the modification of the membrane substrate and surfaces and the incorporation of additives and fillers can contribute significantly to a reduction in fouling.

Surface modification is the simplest technique for modifying the top effective layer of reverse osmosis membranes. Surface modification can change the most relevant surface properties, like the hydrophilicity of the membrane and its roughness, and provide antifouling properties that enhance membrane performance [226]. The surface modification by cross-linkers has been proven effective. The exposure of a commercial thin-film composite reverse osmosis membrane to poly (ethyleneglycol) diacrylate (PEGDA) and ethylene glycol dimethacrylate (EGDMA) after activation with sodium hypochlorite increased both its permeability and rejection and extended the effective lifetime of the membrane, reducing organic fouling [227]. The water-flux decline of the virgin membrane was 62.5% after 10 h of operation, but the PEGDA- and EGDMA-modified membranes showed 43.6% and 53.3% reductions, respectively. The grafting of polyvinyl alcohol through cross-linking with glutaraldehyde to a thin-film composite reverse osmosis membrane was applied to minimize fouling [228]. The performance of the unmodified and modified membranes after exposure to an *E. coli* bacteria cell suspension for 30 min was investigated, and the modified membrane exhibited higher water flux (34 vs. 36 L/m^2^·h).

The development of new membrane materials for reverse osmosis membranes has been deeply emphasized in the last decade. Zwitterionic polymers have attracted wide interest due to their unique properties of containing both cationic and anionic groups while maintaining electroneutrality and high hydrophilicity. These zwitterionic polymers have been utilized as coating materials or grafted layers not only on the surfaces of porous membranes but also as thin-film composite membranes [229,230]. For instance, a thin-film composite polyamide for reverse osmosis was modified to incorporate inner and outer zwitterion-like layers [231]. While the outer zwitterion layer included L-arginine molecules grafted on the polyamide, the inner layer was constituted by the cationic surfactant benzalkonium chloride and the negative charges from the carboxylic acid groups in polyamide. The obtained membranes showed excellent antifouling properties: the flux decline rate was below 40%, and the flux recovery ratio was above 98%. Other engineered functional polymers, like neutral polymers, dendritic polymers, polyelectrolytes or advanced biopolymers, have been tested to achieve improved antifouling properties of reverse osmosis membranes [232]. For example, the grafting of bioinspired N-oxide-based zwitterionic polymer brushes to a commercial polyamide thin-film composite membrane was investigated [233]. Whereas the introduction of the bioinspired N-oxide layer did not significantly affect the membrane performance in both terms of permeability and rejection, improved antifouling properties appeared. The flux decline of the modified membrane was lower (32.1%) and fouling reversibility higher (18.6%) than the virgin membrane (45.4% flux decline and 3.3% fouling reversibility).

Additives and fillers can play an important role in increasing membrane fouling resistance in reverse osmosis membranes. Nanomaterials with desirable properties, such as reasonable particle sizes, low tendency to aggregate, high density of hydrophilic functional groups and good compatibility with polymers, are promising in developing antifouling membranes [225,234,235,236]. Metal–organic frameworks possess convenient properties for being employed as membrane fillers. The incorporation of negatively charged MIL-101(Cr)-Pyz-SO3H nanoparticles into a reverse osmosis membrane produced smoother surfaces and increased the membrane hydrophilicity [237]. The fouling resistance of the modified membrane was assessed by the filtration of a humic acid solution for 48 h, and the reduction in the permeate flux was significantly lower than that of the virgin membrane since the modified membrane maintained more than 90% of the initial flux while the virgin membrane only maintained less than 60%. The photocatalytic nature of TiO_2_, able to produce reactive oxygen species under radiation, has been applied to improve the antifouling characteristics of reverse osmosis membranes [238]. Compared to a virgin membrane, the modified membrane with TiO_2_ nanoparticles experienced lower flux reduction and a higher flux recovery ratio after cleaning, as it increased from 87% for the virgin membrane up to 99% in the modified one.

#### 3.3.2. Nanofiltration

Nanofiltration membranes are nanoporous (the typical pore size ranges from 0.1 to 10 nm), and traditionally, these membranes are constructed from polymers with either positive or negative charges on their surfaces. This characteristic aids in the discrimination of heavy metals and improves membrane performance by fostering electrostatic interactions between the membrane and metal ions. Therefore, the rejection mechanisms of the nanofiltration membranes depend not only on the size exclusion but also on other mechanisms such as Donnan exclusion and dielectric exclusion [239,240,241]. Moreover, hydration mechanisms play a relevant role in rejecting metal ions in different forms [242,243]. The main advantages of nanofiltration are the lower energy requirements (and the corresponding lower operation costs) than reverse osmosis and the high effectiveness for heavy metal removal, with rejection percentages above 90% for some multivalent metal ions [16]. For instance, an innovative asymmetrically charged polyamide nanofiltration membrane exhibited high permeability (more than 19 L/m^2^·h⋅bar), while the rejection percentages for Cu^2+^, Ni^2+^, Zn^2+^ and Pb^2+^ were 93.3%, 93.0%, 91.8% and 88.3%, respectively [244]. Nevertheless, the nanofiltration efficiency depends on several design and operation conditions, such as pressure, temperature, pH, membrane configuration and feed concentration. The effects of all these conditions have been deeply investigated, and optimization of design and operation parameters has been performed [245,246].

Membranes made of inorganic materials have received more attention due to their mechanical, chemical and thermal stability. The primary inorganic membrane materials include metal oxides like titania, zirconia, silica and alumina [247]. A silica nanofiltration membrane synthesized using 3,3,3-trifluoropropyltrimethoxysilane as a precursor was tested to assess its performance with monovalent and bivalent cations and anions [248]. Sulfate was the most effectively retained ion, but under optimal conditions, even NaCl was successfully rejected (91.0% rejection percentage). However, pore size fine-tuning towards the nanofiltration range is difficult and expensive to attain in inorganic membranes and ceramic–polymeric hybrids have been developed considerably since they combine the resistance of a ceramic support with the tunable pore size of a polymeric functional layer [249]. The grafting of organic monomers, such as organophosphonic acids or organosilanes, to ceramic supports is a viable option for producing these hybrid membranes with extended durability and satisfactory long-term performance even in aggressive media [250].

Nevertheless, much more research efforts have been focused on the potential of advanced nanomaterials integrated into polymeric membranes, such as graphene oxides, metal–organic frameworks, carbon nanotubes and MXenes [251,252,253,254,255,256,257,258,259]. While the creation of effective nanofiltration membranes for wastewater treatment has revealed the potential benefits of incorporating organic, inorganic and hybrid nanofillers into a host membrane matrix, it is important to note that these nanofillers may also yield some negative effects. For instance, the presence of nanoparticles may impede the flow of water, reducing the membrane permeability; inadequate interactions between polymeric matrix and nanoparticles may result in the creation of flaws and non-selective pores, decreasing the selectivity; the presence of the nanoparticles may interfere with and reduce the packing of the polymer chains, creating additional void volumes that may reduce the rejection of solutes or the irregular deposition; and the agglomeration of the nanoparticles on the membrane surface or inside the matrix may result in low separation performance [110]. Therefore, a complete understanding of the mechanisms involved in the interactions between the fillers and the polymer matrix is critical [260]. The incorporation of nanofillers (carboxylated cellulose nanocrystals, multi-walled carbon nanotubes and graphene oxide nanoparticles) during the synthesis of polyamide nanofiltration membranes caused semi-crystalline cross-linked structures with increased chain length and free volume. The analysis of the performance of these membranes by molecular dynamics simulation revealed that the growth of free volume caused by the presence of the nanofillers allowed water molecules to readily penetrate the spaces between polymer chains, resulting in enhanced permeability. In addition, the modified crystalline structures influenced the dielectric properties of the membranes and the water confined in the pores, leading to the enhancement of dielectric exclusion and increased separation performance. Therefore, these new fillers can overcome the main limitations of nanofiltration membranes regarding permeation and retention and improve their mechanical, thermal and chemical resistance, but the costs associated with these new materials still must be reduced for them to be economically competitive [107].

Although numerous studies on the improvement of nanofiltration membranes for heavy metal removal have reported positive outcomes, long-term stability remains a challenge. The fouling tendency is clearly a major drawback of wastewater treatment processes based on nanofiltration. Wastewater often contains complex substances that must be separated before contact with the nanofiltration membranes in order to protect them from fouling and allow a longer effective lifetime [261]. Among the different possibilities for pretreatment systems, microfiltration emerges as the most compatible with nanofiltration, especially to avoid particle fouling [262,263]. Optimal pretreatment conditions must be identified for each specific application, and the design of the complete treatment process must be adapted to the defined targets [264]. Unfortunately, pretreatment is not able to completely avoid membrane fouling, so minimization of fouling by the synthesis of membranes with enhanced antifouling properties has been investigated. Membrane modification has been the most frequent method applied to reduce fouling [265]. Tailoring the antifouling properties of nanofiltration membranes to efficiently extend the effective lifetime of membranes while removing heavy metal ions from wastewater has been proven to be an adequate option. Modified interfacial polymerization [266,267,268], grafting techniques [269,270,271,272] and the addition of nanoparticles [273,274,275,276,277] have improved the fouling resistance of nanofiltration membranes. For example, the simultaneous incorporation of metal–organic frameworks (ZIF-8) and graphene oxide in a nanofiltration membrane resulted in enhanced permeability with competitive rejection percentages and provided additional antifouling properties [278]. Under the optimal formulation, the flux decline was negligible during the experimental filtration time, which was consistent with the fact that membrane hydrophilicity increased with the ZIF-8 loading, minimizing the deposition of organic matter on the membrane surface. However, once again, although the long-term performance of these membranes is excellent, they are too expensive compared with commercial membranes. Hence, it is important to realize the economic competitiveness of these improved membranes and identify their optimal potential applications.

More research towards the integration of renewable energy with nanofiltration processes should also be implemented since it will result in more cost-efficient systems with lower environmental impacts [279]. Decentralized nanofiltration systems that combine the use of photovoltaic and wind energy have been designed and tested to treat brackish water successfully [280]. The stability of electrical and hydraulic conditions and constant retentions of solutes allowed the correct performance of the process, and the quality of the permeate stream produced by the plant confirmed the feasibility of the coupling of the mixed renewable energies with the nanofiltration treatment [281]. Therefore, the consideration of solar and wind sources allows the operation of decentralized systems even in remote areas [282], thus promoting the implementation of sustainable solutions based on renewable energy [283].

#### 3.3.3. Ultrafiltration

Ultrafiltration membranes show permeability values greatly higher than nanofiltration membranes and lower operation pressure, but they are considered too loose for the removal of dissolved metallic ions. This advantageous permeability and low pressure requirements can be leveraged for heavy metal removal by means of the use of auxiliary chemicals that can interact with dissolved metal ions (micellar-enhanced and complex-enhanced ultrafiltration) or the incorporation of adequate fillers that can adsorb these ions (adsorptive ultrafiltration) [33].

Micellar-enhanced ultrafiltration holds significant potential for the efficient removal of heavy metal ions from wastewater. This technique combines the principles of ultrafiltration with the use of surfactant micelles to enhance the separation process [28]. A surfactant, typically anionic or cationic, is introduced into the metal-polluted wastewater, and its molecules self-assemble into micelles, which are tiny, spherical structures with hydrophobic cores and hydrophilic exteriors. These micelles can effectively trap heavy metal ions within their hydrophobic cores, and an ultrafiltration membrane is employed to separate the micelles, along with the entrapped heavy metal ions. Heavy metal ions, such as lead, cadmium, copper and nickel, have been successfully retained by this approach [284,285]. One critical aspect of micellar-enhanced ultrafiltration is the selection of the surfactant, which must be effective for the removal of the specific heavy metals present in wastewater and present a low critical micelle concentration, since micelles are not formed until this surfactant concentration is achieved. After adequate selection, even trace amounts of heavy metals can be effectively removed from wastewater streams [286]. Additionally, the recovery of the heavy metals from the micelles can be carried out, promoting the potential reuse or recycling of these valuable resources. This approach has been applied to the recovery of rare earths [287]. Sodium dodecyl sulfate (SDS) was combined with 20 and 150 kDa ultrafiltration membranes to separate rare earth elements from synthetic and real leachates. The system was operated just under 1 bar pressure with high permeability (higher than 50 L/m^2^·h⋅bar), and the rejection percentages for rare earths were between 84.6% and 99.3% with an optimal SDS dose. Subsequent post-treatment (two-step precipitation using CaCl_2_ and Na_2_CO_3_) of the retentate from the micellar-enhanced ultrafiltration stage allowed the recovery of more than 90% of the rare earths, with values as high as 99.65%. Nevertheless, the proposed process was not selective for rare earths in the presence of other cations, and pretreatment should be considered before the membrane stage.

The main limitation of micellar-enhanced ultrafiltration is the potential for surfactant contamination in the treated water. Residual surfactants can permeate and persist in the treated water, which may be undesirable [288,289]. This could lead to concerns about the introduction of additional pollutants with potential ecological or health implications, implying the need for post-treatment steps to remove or neutralize these surfactants. Therefore, the consideration of biodegradable surfactants, such as rhamnolipids, has been proposed [290,291,292]. Another biosurfactant, surfactin, has been also tested for heavy metal removal by micellar-enhanced ultrafiltration [293]. The addition of this surfactant in a 3:1 ratio to the metals allowed the removal of over 98% of the metals (Cr, Pb, Cd and Co) by an ultrafiltration membrane with a pore size of 50 nm. Adequate membrane cleaning using acid and alkaline solutions kept the membrane operative for at least four consecutive cycles with a flux reduction lower than 10%. Additionally, the presence of surfactants can reduce drastically the permeability of a membrane [294,295]. Furthermore, this technique can be sensitive to variations in operation conditions and the nature of the target heavy metals [296]. Factors like pH, temperature and the presence of other ions in the solution can influence the efficiency of micelle formation and heavy metal removal [297]. Consequently, careful optimization and monitoring may be required to achieve consistent and reliable results, making this option potentially less robust than some other alternative options for heavy metal removal [298]. Lastly, the choice of a surfactant is critical and must be tailored to the specific heavy metal contaminants, adding complexity and cost to the process [299].

Complex-enhanced ultrafiltration is a promising option for addressing the removal of heavy metal ions from wastewater. This filtration technique combines the advantages of ultrafiltration with the selectivity of chelating agents or complexing agents to efficiently capture and remove heavy metal ions. It relies on the formation of stable complexes between metal ions and specific chelating agents, such as organic ligands or polymers [300]. These chelating agents possess a high affinity for the target metal ions, forming strong, reversible bonds. When the complexed solution is passed through an ultrafiltration membrane, the metal complexes, with sizes larger than the diameter of the membrane pores, can be effectively retained. Operating parameters, like the applied pressure, cross flowrate, pH of the feed solution and molar ratio of the chelating agent to metal, have been studied to investigate the maximal effectiveness of the process for the selective separation of different metals [301,302]. One of the key advantages of complex-enhanced ultrafiltration is its ability to target specific heavy metal ions, making it highly selective in its removal process. Traditional chelating agents like EDTA (ethylene diamine tetraacetic acid) or DTPA (diethylenetriamine pentaacetic acid) form stable complexes with heavy metal ions, preventing their presence in the permeated stream [142,303]. Other polyelectrolytes, like polyacrylic acid, polyethylenimine, chitosan and polystyrenesulfonate, have been successfully employed for this application too [304,305]. The selectivity of these agents reduces the risk of interfering with other present ions. In addition, this technique has the potential to contribute to sustainable practices in wastewater management by allowing the recovery of complexed heavy metals from wastewater [306]. The recovery of rare earths is a great example. The employment of phosphonic chitosan (PCS) as a complexing agent was useful for retaining several rare earth elements (La, Ce, Dy, Yb and Y) by means of a 10 kDa polyether sulfone ultrafiltration membrane [307,308]. Under the optimal pH (values above 5) and complexing agent concentration, rejection percentages above 99% were achieved. Moreover, shear-induced dissociation of the metallic complexes allowed the selective recovery of the different rare earths, even in the presence of other cationic metals, such as Ca.

Membrane fouling must be mentioned as the most important drawback when complex-enhanced ultrafiltration is evaluated. As the chelating agents form stable complexes with heavy metal ions, the deposition of solid particles or gel-like substances on the membrane surface can occur [309]. Moreover, the added chemicals can also interact with other species present in the feed solution, such as organic matter or particulate contaminants. This can lead to the accumulation of fouling materials on the ultrafiltration membrane surface, reducing its permeability and efficiency over time. To mitigate this issue, frequent membrane cleaning and maintenance are required, which can increase operational costs. The selection of the most appropriate operating conditions can reduce membrane fouling. A complex-enhanced ultrafiltration system based on humic acid for forming complexes with Mn was subject to optimization in order to minimize fouling. The operation in sub-critical flux conditions (applying pressure no higher than 1 bar) extended the effective lifetime of the membrane, which was made of polyethersulfone and had a molecular cut-off of 30 kDa. Although a higher pH improved Mn rejection (the maximal rejection corresponded to pH 11), the operation at pH 7 reduced membrane fouling (lower than 30% flux decline versus higher than 40% at pH 11) and maintained a rejection percentage of above 90%. As expected, higher complexing concentrations increased membrane fouling. Another notable drawback is the selectivity of the chelating agents used. While these agents are designed to have a high affinity for specific heavy metal ions, they may not be equally effective for all types of metal contaminants. In real applications, wastewater streams often contain a mixture of heavy metal ions with varying properties and concentrations. In addition, other chemical species are also present, so achieving optimal selectivity for each metal ion can be challenging, and there is a risk of incomplete removal [310,311]. Additionally, the cost and availability of suitable chelating agents can be limiting factors, as some highly effective agents may be expensive or environmentally problematic, further complicating the practical implementation of complex-enhanced ultrafiltration for heavy metal ion removal.

Adsorptive ultrafiltration combines the principles of ultrafiltration with the adsorption capacity of specialized adsorbents, and it can be applied to the removal of heavy metal ions in aqueous solutions. The process involves passing the contaminated water through a porous ultrafiltration membrane where adsorbent particles have been embedded [46]. These adsorbents are designed to possess a high affinity for heavy metal ions and be compatible with the membrane matrix, enabling them to selectively bind and remove these metal ions as they pass through the membrane pores [312]. Adsorptive ultrafiltration offers a high removal efficiency, capable of achieving stringent regulatory standards for heavy metal discharge. Carboxylated cellulose nanocrystals as adsorbents were incorporated into a bioinspired ultrafiltration membrane made of bacterial cellulose coated with polydopamine [313]. This adsorptive membrane was effective for multiple contaminants, including heavy metals, organic dyes and natural organic matter. Regarding heavy metals, lead was removed from an initial concentration equal to 30 mg/L with rejection percentages close to 100% during the first hour of testing, and after 6 h of filtration, the rejection was still higher than 60%. Cadmium and copper also were successfully retained by the membrane, following a preference that was contrary to the radius of the corresponding hydrated ions. Adsorptive ultrafiltration is a versatile technology that can be applied to various types of wastewaters, making it suitable for a wide range of applications. Additionally, adsorbent materials can be tailored to specific heavy metal contaminants, providing a tailored selective approach to water treatment. The adsorbents can often be regenerated, offering a sustainable and cost-effective solution for heavy metal ion removal. The effects of pH, metal ion concentration, adsorbent content, temperature and applied pressure on the treatment efficiency and permeate flux have been investigated [314].

However, there are certain drawbacks associated with adsorptive ultrafiltration. One significant limitation is the reduced permeability of the ultrafiltration membranes due to the presence of adsorbent particles in their structure [315]. In other cases, the presence of the nanofillers can produce void volumes that enhance the solvent permeation but also increase the passage of solutes through the membrane [316]. Furthermore, the adsorption capacity of the sorbent material is finite, and once it reaches its saturation point, the system requires regeneration or replacement of the adsorbents, which can be costly and require harsh chemicals [115,317]. Additionally, the presence of other competing ions in the wastewater can interfere with the selectivity and efficiency of adsorption, necessitating pretreatment or further process optimization [313]. These challenges underscore the need for ongoing research and development to enhance the reliability and efficiency of adsorptive ultrafiltration for heavy metal ion removal in wastewater treatment applications [318].

#### 3.3.4. Microfiltration

Microfiltration is the pressure-assisted membrane separation process with the largest pore size, and it serves as an adequate technology for removing particles and colloids. Microfiltration employs porous membranes with pore sizes typically ranging from 0.1 to 10 µm and allows smaller molecules like water and dissolved ions to pass through while blocking the passage of larger particles, such as suspended solids, bacteria and other microorganisms. Microfiltration has emerged as a promising pretreatment technology for the removal of heavy metals before employing more advanced techniques like nanofiltration and reverse osmosis [94,95]. One of the key advantages of microfiltration is its ability to effectively remove suspended particles and colloids, thus preventing fouling and clogging in downstream processes [319]. This is crucial in heavy metal removal when these contaminants appear as suspended forms or precipitates, along with other particulate matter [320,321,322]. Furthermore, microfiltration enhances the overall efficiency of heavy metal removal processes. By reducing the particle load in the water, it allows nanofiltration and reverse osmosis membranes to focus on the selective removal of heavy metals without being interfered with by the presence of other impurities [323]. This results in improved permeate quality and a longer effective lifetime for the membranes. Additionally, microfiltration is a cost-effective pretreatment method, as it requires low energy consumption and maintenance compared to alternative pretreatment options. The use of microfiltration without additional technologies has demonstrated that can fulfill the requirements of providing a high-quality feed to nanofiltration and reverse osmosis stages from different raw water qualities [324]. Nevertheless, although porous microfiltration membranes are not adequate for the removal of dissolved heavy metals, the employment of auxiliary chemicals can be useful, as in the case of ultrafiltration membranes.

Micellar-enhanced microfiltration is an innovative and efficient technique used for the removal of heavy metals from aqueous media, combining the principles of microfiltration and surfactant chemistry to enhance the separation of heavy metal ions. A surfactant, typically a micelle-forming compound, is added to form micelles in the solution, which are tiny aggregates with hydrophilic heads and hydrophobic tails that interact with the heavy metals [325]. The heavy metal ions bind to the hydrophobic regions of these micelles, effectively creating larger suspended complexes that are much larger in size than the original metal ions, making them separable by microfiltration membranes, since the surfactant-bound heavy metal complexes are often larger than the membrane’s pore size. This way, metal rejection percentages above 85% can be achieved [326], with maximal values above 99% for specific cases [120]. For instance, a positively charged microporous membrane was prepared to remove hexavalent chromium from wastewater using stearyltrimethyl ammonium chloride (STAC) as a surfactant [327]. The process attained 99.8% rejection combined with a permeability of 100 L/m^2^·h·bar under optimal conditions. The influence of the most relevant operation variables (such as applied pressure, feed flowrate, surfactant type and concentration, pH and background electrolytes) on the performance of micellar-enhanced microfiltration has been deeply investigated, considering the variation in metal and surfactant rejections and the corresponding permeate flux [328,329,330].

Micellar-enhanced microfiltration offers several advantages, including low energy consumption and the ability to effectively remove a wide range of heavy metal contaminants from aqueous solutions. Nevertheless, one significant limitation is the potential for surfactant contamination in the treated water. To achieve effective complex formation and enhanced removal, relatively high surfactant concentrations are required. These surfactants may not be completely removed from the treated water, as surfactant rejection percentages below 30% have been reported [331], which can lead to unintended consequences, including environmental concerns and altered water quality. Moreover, despite the increase in metal rejection with the addition of a higher quantity of surfactant, a considerable decrease in steady-state permeate flux is a major drawback of this process [332]. This flux decline is strongly dependent on the surfactant type. Additionally, the surfactant molecules used in micellar-enhanced microfiltration may not be suitable for all heavy metal removal applications, as their efficiency can vary depending on the specific metal ions present, potentially limiting their versatility in addressing various heavy metal contaminants simultaneously.

Complex-enhanced microfiltration is another advanced method employed for the efficient removal of heavy metal ions using microfiltration membranes. This technique involves the use of chemical chelating ligands that selectively bind with heavy metal ions to form large insoluble complexes [126]. These complexes are larger in size compared to individual heavy metal ions and are less likely to pass through microfiltration membranes. The choice of water-soluble complexing ligands remains important for the development of this technology [333], although the grafting of these ligands to membrane surfaces was also successful [122]. The functionalization of a PVDF membrane with 8-hydroxyquinoline groups is an illustrative example [121]. This membrane was able to remove dissolved cadmium and nickel from aqueous solutions. The complexing process was more efficient at higher pH conditions, and the membrane showed a preference for cadmium in the presence of both metals, attaining rejection percentages of around 60%. The influence of the most relevant operation variables (such as ligand type and concentration, pH, wastewater composition, applied pressure and feed flux) on the performance of complex-enhanced microfiltration has been deeply investigated, considering the variation in metal and surfactant rejections and the corresponding permeate flux [123]. Nevertheless, taking into account the complexity of the chelating–microfiltration process and the mutual dependence of the different design and operation parameters, non-physical models as the ones derived from artificial neural networks have been preferred to represent the process performance [334].

Complex-enhanced microfiltration offers several advantages in heavy metal removal processes. Firstly, it can be highly effective in capturing a wide range of heavy metal ions thanks to the strong binding affinity of the chelating ligands [335]. Secondly, this technique minimizes the direct contact of heavy metal ions with the membrane surface, reducing scaling and the appearance of incrustations on the membrane surface. Moreover, it can be tailored to target specific heavy metal contaminants, making it a versatile solution for various water treatment applications. However, it also has limitations, with one key drawback being the potential for ligand contamination. Similar to micellar-enhanced microfiltration, complex-enhanced microfiltration relies on the addition of auxiliary chemicals (chelating ligands) to form large complexes with heavy metal ions. If not managed carefully, these ligands can remain in the treated water, potentially introducing new chemical compounds. In addition, although metal rejection percentages are clearly favored by high concentrations of the chelating complexes, achieving values above 95% [336], the presence of the chelating ligands can drastically reduce the permeate flux. Furthermore, this process may not be cost-effective for treating water with low concentrations of heavy metals, as it relies on the formation of strong chemical bonds between ligands and heavy metal ions, which may be less efficient at lower metal concentrations.

Adsorptive microfiltration represents an intensified approach for the removal of heavy metals from contaminated water sources, since this technology combines the principles of microfiltration with adsorption processes, taking advantage of the adsorptive capacity of specialized porous membranes. These membranes are typically functionalized with specific adsorbent materials or coatings that have a high affinity for heavy metal ions [337,338]. Nanomaterials, such as metal oxides, metal–organic frameworks, zeolites and carbon-based materials, have attracted much attention as adsorptive fillers in microfiltration membranes due to their high active surface area, large number of functional groups and high chemical and thermal stability [130]. When contaminated water flows through these membranes, heavy metal ions are attracted to the adsorbent surfaces and selectively bind to them. This process efficiently separates heavy metals from the water stream, preventing them from passing through the membrane. The rejection percentages of these adsorptive membranes for heavy metal ions can reach values above 94%, displaying a fine rejection and removing performance [339,340,341]. The influence of the main parameters, such as pH, applied pressure and concentration of heavy metals, on the removal efficiency of adsorptive membranes has been evaluated [342].

Adsorptive microfiltration offers several notable advantages for heavy metal removal. Firstly, it provides a dual mechanism for purification by combining microfiltration’s particle removal capability with adsorption’s affinity for heavy metals. This results in an efficient treatment process that can handle a broad spectrum of coexisting heavy metal contaminants [343]. Secondly, it is particularly effective in treating water with low heavy metal concentrations, as it can capture even trace amounts of these pollutants when selective adsorbents are selected. Additionally, these systems are often designed to be regenerable, allowing for the recovery and recycling of heavy metals, which can be economically beneficial and environmentally responsible. But adsorptive microfiltration has its own set of drawbacks, including the potential for membrane fouling. The presence of the adsorbents within the membrane structure can reduce the permeability, and as heavy metal ions adsorb onto the membrane surface or within the adsorbent layer, the accumulation of adsorbates can lead to reduced permeability, making frequent membrane cleaning or replacement obligatory [127]. This fouling issue can significantly increase operating costs. Additionally, the choice of adsorbent materials is critical, as different materials have varying affinities for specific heavy metal ions, potentially limiting the technology’s effectiveness for certain contaminants if not chosen appropriately [344]. The regeneration of the adsorption capacity of a membrane requires additional tasks that require the use of auxiliary chemicals [345].

## 4. Conclusions

A comprehensive overview of research on pressure-driven membrane technologies for the removal of heavy metals from wastewater was compiled based on the findings of a bibliometric analysis. This analysis included information on annual publications, document types, languages, countries, institutions, categories, journals and keywords. The accumulated publications on this topic exhibited exponential growth over the examined period (1972–2023), indicating an intensified interest in this field, particularly since the last decade of the 20th century. China emerged as the most prolific country in terms of the total number of publications, with India and the USA following closely behind. Notably, Chinese institutions featured prominently among the most productive ones. The study identified *Environmental Science* as the most prevalent knowledge category, followed by *Chemistry*, *Chemical Engineering*, *Engineering* and *Materials Science*. Therefore, the multidisciplinary approach to the application of pressure-driven membranes for heavy metal removal from wastewater considers the environmental consequences of these types of metals in natural water bodies, the technical solutions for avoiding the presence of these types of metals in natural water bodies, and the scientific fundamentals for improving the existing materials and develop new ones to obtain advanced membranes with improved performance. The detailed analysis of the most frequent keywords and the corresponding bibliometric network analysis confirmed the higher relevance of low-pressure technologies (microfiltration and ultrafiltration) when compared to high-pressure technologies (nanofiltration and reverse osmosis).

Although reverse osmosis membranes presented the highest removal percentages (achieving values above 99%) for heavy metals, this technology was not preferred in most cases because of the higher energy consumption due to high increased operation pressure gradient and the elevated maintenance costs attributable to the regeneration of the membranes due to fouling. Reverse osmosis only appeared as a promising option when high-quality permeate was desired, the initial heavy metal content was very high or there was the possibility to recover high-value metals. The research on new reverse osmosis membranes was mainly focused on increasing permeability and improving resistance to fouling. The consideration of renewable energy could reduce the environmental impact of the reverse osmosis processes. Nanofiltration maintains adequate rejection performance (values above 90%, especially for multivalent ions, which are close to the ones obtained by reverse osmosis) and can be operated under lower pressure requirements than reverse osmosis. Significant research efforts have been applied to improve the permeability of the membrane in order to increase the permeate production, and new materials are called to play a relevant role in this target. Although inorganic membranes based on ceramic materials have gained increasing interest, the investigation related to the incorporation of nanomaterials (such as metal–organic frameworks, carbon nanotubes or graphene particles) as fillers in polymeric nanofiltration membranes was more relevant. The fouling of nanofiltration membranes was a very important aspect to be considered, and new efforts have been applied to extend the effective lifetime in the long term while maintaining adequate permeate production and metal rejections. As in the case of reverse osmosis, nanofiltration systems designed to remove heavy metals have been successfully operated using renewable energy directly. Ultrafiltration is based on porous membranes that cannot retain dissolved heavy metals, but the use of auxiliary chemicals to form metallic complexes or micelles that can be retained by this type of membrane was demonstrated to be a valid approach. Moreover, the incorporation of embedded adsorbents within the ultrafiltration membrane structure has been deeply investigated. Both alternatives can take advantage of the higher permeability of ultrafiltration membranes when compared to high-pressure technologies. This same approach has been applied to microfiltration in order to develop complex-enhanced microfiltration, micellar-enhanced microfiltration and adsorptive microfiltration. Nevertheless, the implementation of microfiltration as an initial pretreatment stage before other membrane technologies was still the most frequent application of microfiltration membranes in processes for removing heavy metals from wastewater.

## Figures and Tables

**Figure 7:**
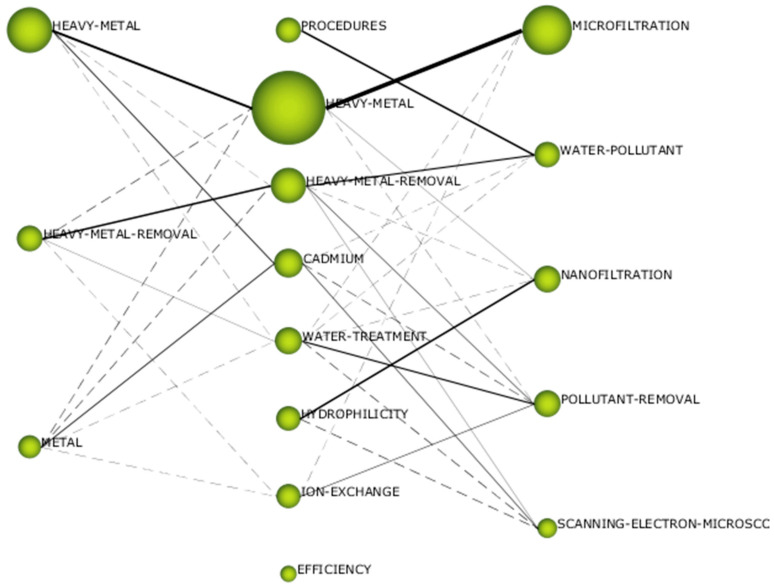
Thematic evolution structure of pressure-driven membranes for heavy metal removal research (1972–2021).

**Table 1:** The languages employed by the publications.

Language	Publications	Contribution (%)
English	2085	94.39
Chinese	61	2.76
German	11	0.50
Russian	10	0.45
Persian	10	0.45
French	7	0.32
Croatian	2	0.09
Japanese	2	0.09
Arabic	1	0.05
Lithuanian	1	0.05
Polish	1	0.05
Portuguese	1	0.05
Spanish	1	0.05
Undefined	16	0.72

**Table 2:** The top 14 most productive countries (at least 60 documents).

Country	Publications	Contribution (%)
China	471	21.36
India	356	16.15
United States	213	9.66
Iran	161	7.30
Malaysia	94	4.26
United Kingdom	76	3.45
Spain	72	3.27
Germany	70	3.17
Canada	68	3.08
Italy	66	2.99
South Korea	66	2.99
Saudi Arabia	65	2.95
Turkey	62	2.81
France	61	2.77

**Table 3:** The top 11 most productive institutions (at least 20 documents).

Institution	Country	Publications	Contribution (%)
Ministry of Education (China)	China	51	2.31
Chinese Academy of Sciences	China	51	2.31
University Teknologi Malaysia	Malaysia	44	2.00
National University of Singapore	Singapore	28	1.27
Zhejiang University of Technology	China	26	1.18
University of Chinese Academy of Sciences	China	25	1.13
Zhejiang University	China	23	1.04
Razi University	Iran	23	1.04
Harbin Institute of Technology	China	22	1.00
Hunan University	China	20	0.91
Anna University	India	20	0.91

**Table 4:** The top 10 most popular subject categories (at least 100 documents).

Subject	Publications	Contribution (%)
Environmental Science	1145	51.93
Chemistry	798	36.19
Chemical Engineering	770	34.92
Engineering	579	26.26
Materials Science	501	22.72
Biochemistry, Genetics and Molecular Biology	267	12.11
Medicine	136	6.17
Energy	122	5.53
Agricultural and Biological Sciences	117	5.31
Physics and Astronomy	115	5.22

**Table 5:** The top 14 most popular journals (at least 25 documents).

Source	SJR (2022)	IF (2022)	JCI (2022)	Publications	Contribution (%)
*Journal of Membrane Science*	1.910	9.5	1.85	109	4.94
*Desalination*	1.471	9.9	1.93	99	4.49
*Chemosphere*	1.727	8.8	1.55	72	3.27
*Desalination and Water Treatment*	0.267	1.1	0.25	70	3.17
*Separation and Purification Technology*	1.339	8.6	1.37	62	2.81
*Journal of Hazardous Materials*	2.570	13.6	1.93	61	2.77
*Chemical Engineering Journal*	2.803	15.1	1.99	51	2.31
*Water Science and Technology*	0.548	2.7	0.43	51	2.31
*Water Research*	3.338	12.8	2.15	41	1.86
*Membranes*	0.489	4.2	0.61	36	1.63
*Journal of Environmental Management*	1.678	8.7	1.46	29	1.32
*Science of the Total Environment*	1.946	9.8	1.68	29	1.32
*Journal of Environmental Chemical Engineering*	1.198	7.7	0.94	26	1.18
*Separation Science and Technology*	0.470	2.8	0.40	25	1.13

**Table 6:** The top 10 most cited papers.

Ranking	Article	Times Cited
1	Title: Removal of heavy metal ions from wastewaters: A review Author(s): Fu, F., Wang Q. Journal: *Journal of Environmental Management* Year: 2011	6980
2	Title: New trends in removing heavy metals from industrial wastewater Author(s): Barakat, M.A. Journal: *Arabian Journal of Chemistry* Year: 2011	2335
3	Title: Review of technologies for oil and gas produced water treatment Author(s): Fakhu’l-Razl, A., Pendashteh, A., Abbullah, L.C., (…), Madaeni, S.S., Abidin, Z.Z. Journal: *Journal of Hazardous Materials* Year: 2009	1729
4	Title: Physico-chemical treatment techniques for wastewater laden with heavy metals Author(s): Kurniawan, T.A., Chan, G.Y.S., Lo, W.-H., Babel, S. Journal: *Chemical Engineering Journal* Year: 2006	1666
5	Title: Interactions of metal ions with chitosan-based sorbents: a review Author(s): Gulbal, E. Journal: *Separation and Purification Technology* Year: 2004	1640
6	Title: Sustainable technologies for water purification from heavy metals: review and analysis Author(s): Bolisetty, S., Peydayesh, M., Mezzenga, R. Journal: *Chemical Society Reviews* Year: 2019	917
7	Title: Surfactant-enhanced remediation of contaminated soil: A review Author(s): Mulligan, C.N., Yong, R.N., Gibbs, B.F. Journal: *Engineering Geology* Year: 2001	895
8	Title: Removal of heavy metals from industrial wastewaters: A review Author(s): Azimi, A., Azari, A., Rezakazemi, M., Ansarpour, M. Journal: *ChemBioEng Reviews* Year: 2017	831
9	Title: Selective ion penetration of graphene oxide membranes Author(s): Sun, P., Zhu, M., Wang, K., (…), Xu, Z., Zhu, H. Journal: *ACS Nano* Year: 2013	631
10	Title: Microalgae—A promising tool for heavy metal remediation Author(s): Suresh Kumar, K., Dahms, H.U., Won, E.J., Lee, J.-S., Shin, K.H. Journal: *Ecotoxicology and Environmental Safety* Year: 2015	603

**Table 7:** Centrality and density values of the different clusters identified in the bibliometric network analysis.

Cluster	Number of Documents	Centrality	Density
**Subperiod 1 (1972–2010)**			
Heavy Metal	411	140.29	49.30
Heavy Metal Removal	168	108.90	27.35
Metal	133	101.49	13.76
**Subperiod 2 (2011–2020)**			
Procedures	159	250.47	113.32
Heavy Metal	763	353.07	68.82
Heavy Metal Removal	288	300.98	34.80
Cadmium	206	166.74	18.44
Water Treatment	185	119.91	15.63
Hydrophilicity	151	124.70	18.11
Ion Exchange	147	129.78	11.50
Efficiency	49	74.19	4.15
**Subperiod 3 (2021–2023)**			
Microfiltration	461	242.02	80.28
Water Pollutant	165	195.96	64.94
Nanofiltration	180	127.22	33.32
Pollutant Removal	181	170.66	20.33
Scanning Electron Microscopy	91	111.64	12.97

## Data Availability

The authors can provide the employed data on demand.

## Supplementary Materials

The following supporting information can be downloaded at: https://www.mdpi.com/article/10.3390/membranes14080180/s1, Figure S1: Evolution of the thematic network structure of the cluster Heavy Metal (1972–2010), Figure S2: Evolution of the thematic network structure of the cluster Heavy Metal Removal (1972–2010), Figure S3: Evolution of the thematic network structure of the cluster Metal (1972–2010), Figure S4: Evolution of the thematic network structure of the cluster Procedures (2011–2020), Figure S5: Evolution of the thematic network structure of the cluster Heavy Metal (2011–2020), Figure S6: Evolution of the thematic network structure of the cluster Heavy Metal Removal (2011–2020), Figure S7: Evolution of the thematic network structure of the cluster Cadmium (2011–2020), Figure S8: Evolution of the thematic network structure of the cluster Water Treatment (2011–2020), Figure S9: Evolution of the thematic network structure of the cluster Hydrophilicity (2011–2020), Figure S10: Evolution of the thematic network structure of the cluster Ion Exchange (2011–2020), Figure S11: Evolution of the thematic network structure of the cluster Efficiency (2011–2020), Figure S12: Evolution of the thematic network structure of the cluster Microfiltration (2021–2023), Figure S13: Evolution of the thematic network structure of the cluster Water Pollutant (2021–2023), Figure S14: Evolution of the thematic network structure of the cluster Nanofiltration (2021–2023), Figure S15: Evolution of the thematic network structure of the cluster Pollutant Removal (2021–2023), Figure S16: Evolution of the thematic network structure of the cluster Scanning Electron Microscopy (2021–2023).
