# Supplementary material for: Removal of Heavy Metals from Wastewaters and Other Aqueous Streams by Pressure-Driven Membrane Technologies: An Outlook on Reverse Osmosis, Nanofiltration, Ultrafiltration and Microfiltration Potential from a Bibliometric Analysis
Source: Membranes (Basel). 2024 Aug 22;14(8):180. doi: 10.3390/membranes14080180 (PMC11355994; doi:10.3390/membranes14080180)
Supplement: Supplementary file 1 [file membranes-14-00180-s001.zip › membranes-3140230-supplementary.pdf]

REMOVAL OF HEAVY METALS FROM WASTEWATERS AND OTHER AQUEOUS STREAMS BY PRESSURE-DRIVEN MEMBRANE TECHNOLOGIES: AN OUTLOOK ON REVERSE OSMOSIS, NANOFILTRATION, ULTRAFILTRATION AND MICROFILTRATION POTENTIAL FROM A BIBLIOMETRIC ANALYSIS

K. Castro, R. Abejón *

Universidad de Santiago de Chile (USACH), Departamento de Ingeniería Química y Bioprocesos.
Av. Libertador Bernardo O'Higgins 3363, Estación Central, Santiago 9170019, Chile

* Corresponding author Email: ricardo.abejonr@usach.cl

SUPPLEMENTARY INFORMATION

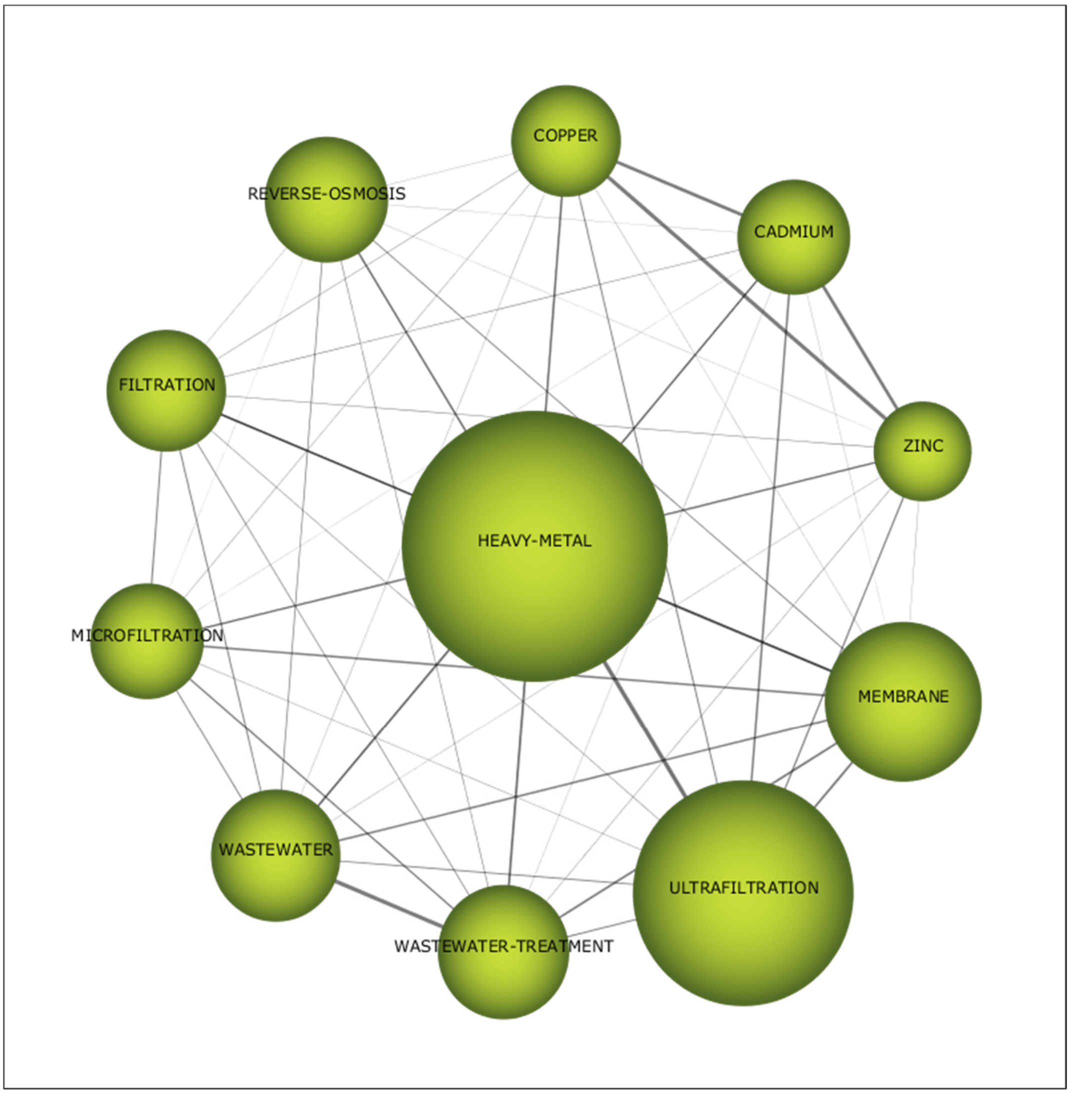

Figure S1. Evolution of the thematic network structure of the cluster Heavy Metal (1972-2010).

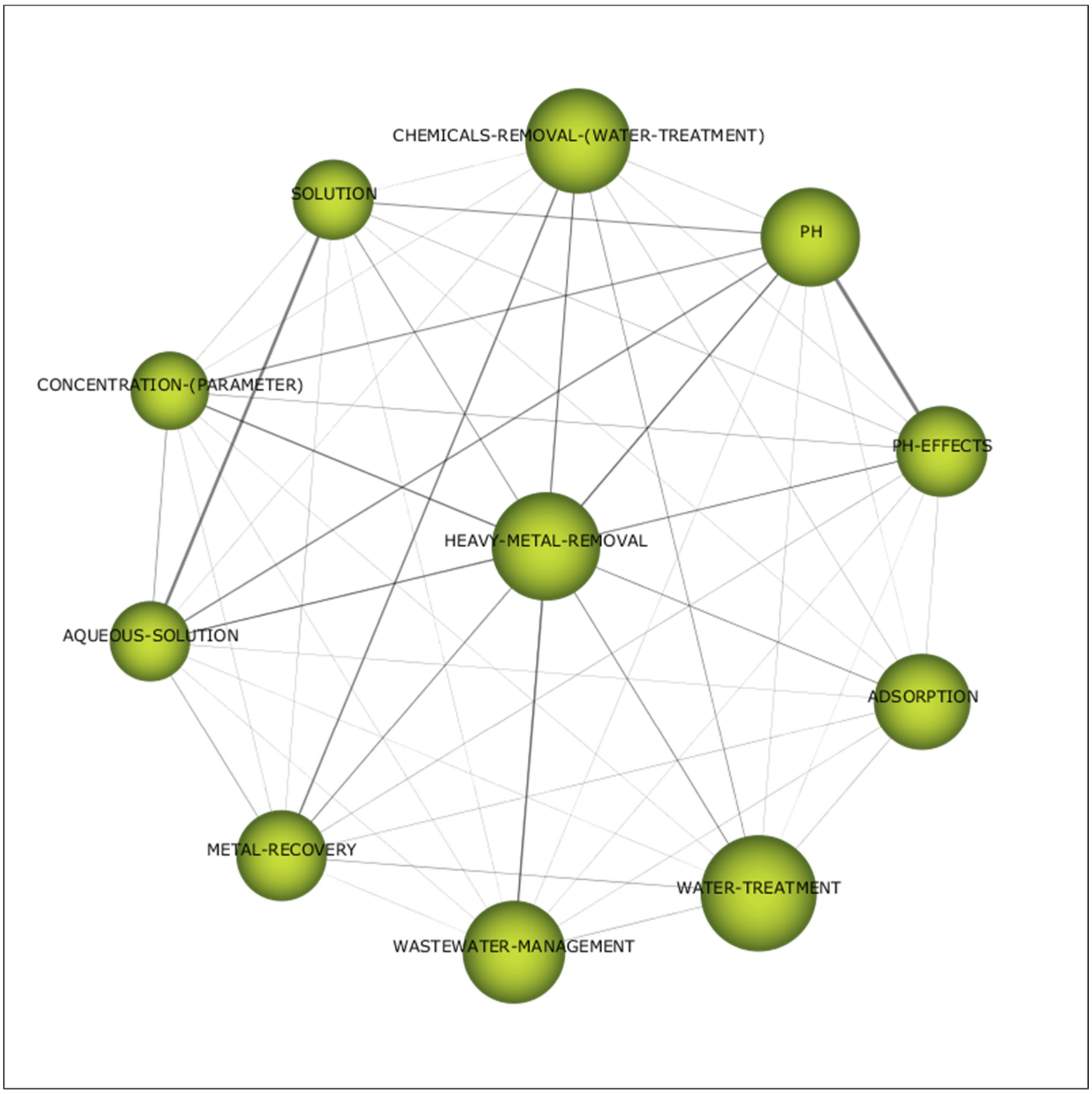

Figure S2. Evolution of the thematic network structure of the cluster Heavy Metal Removal (1972-2010).

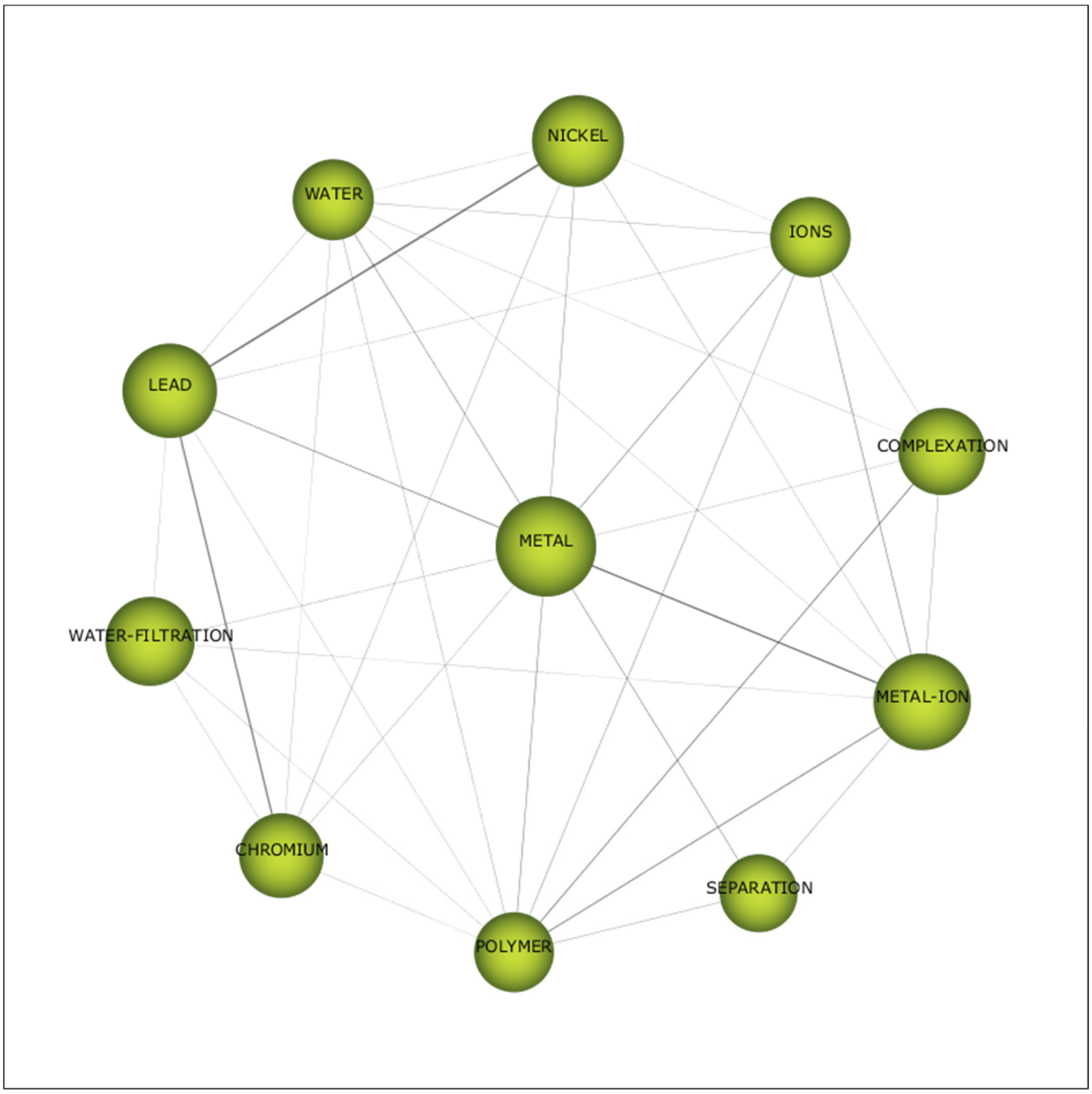

Figure S3. Evolution of the thematic network structure of the cluster Metal (1972-2010).

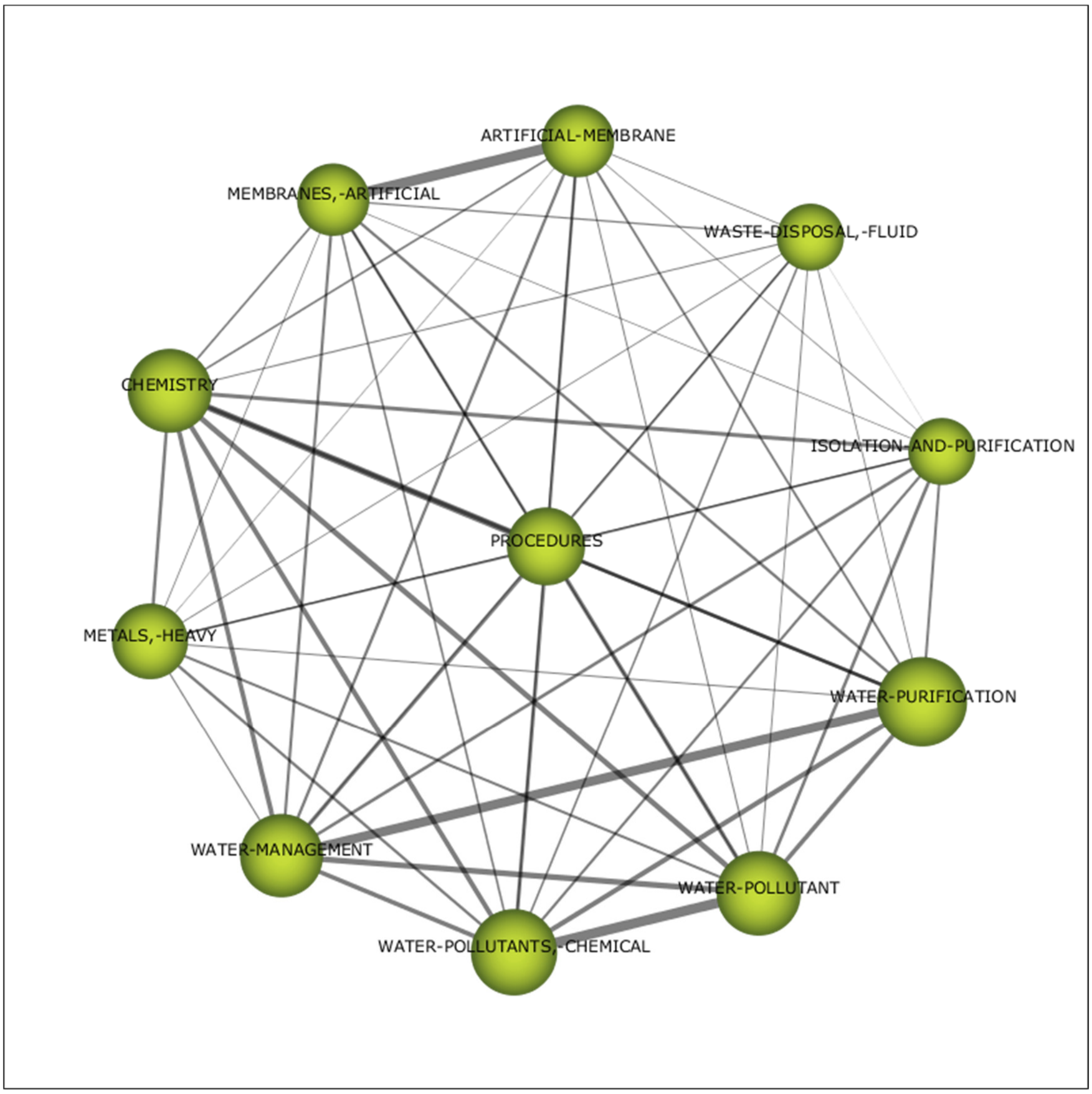

Figure S4. Evolution of the thematic network structure of the cluster Procedures (2011-2020).

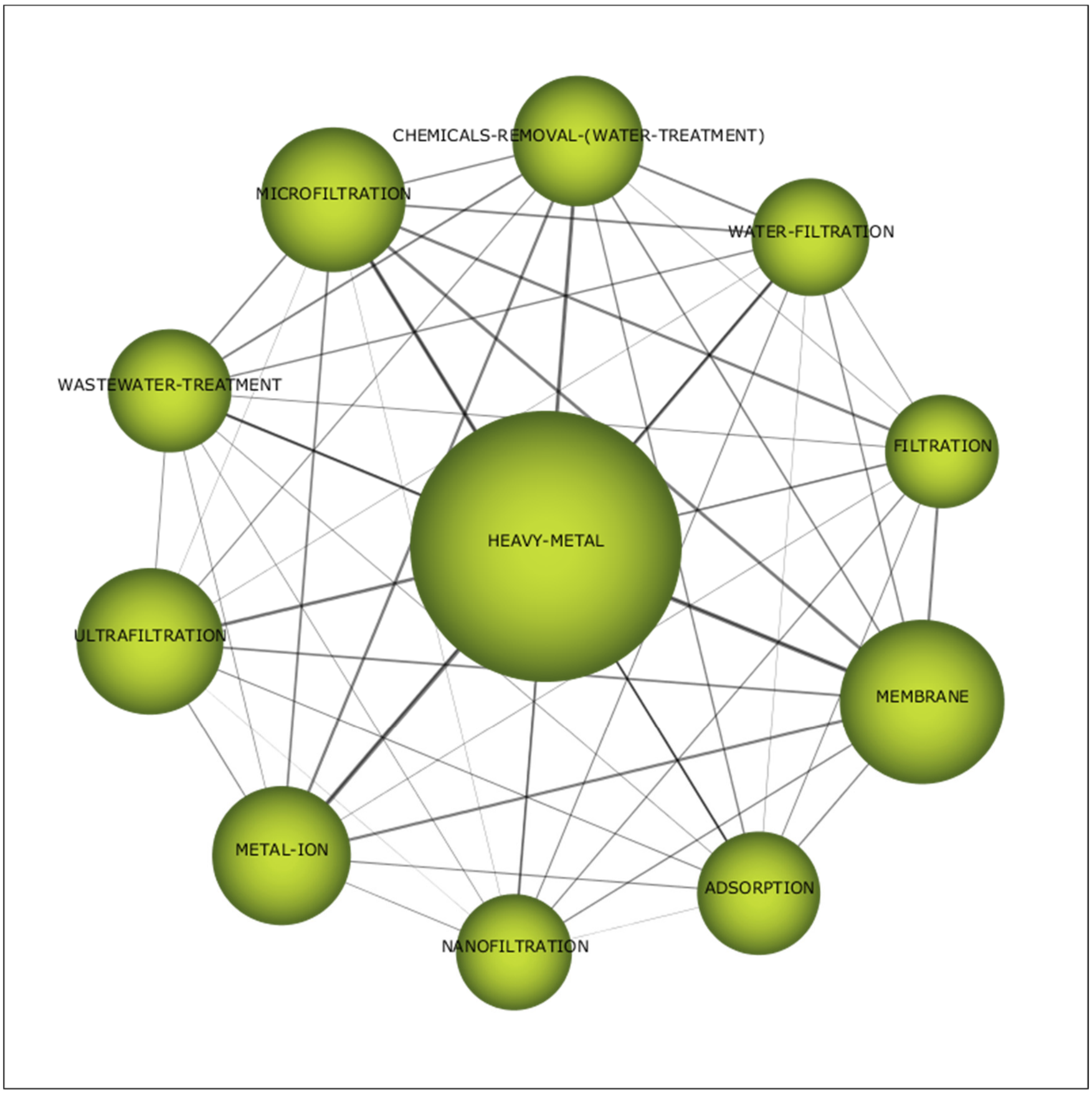

Figure S5. Evolution of the thematic network structure of the cluster Heavy Metal (2011-2020).

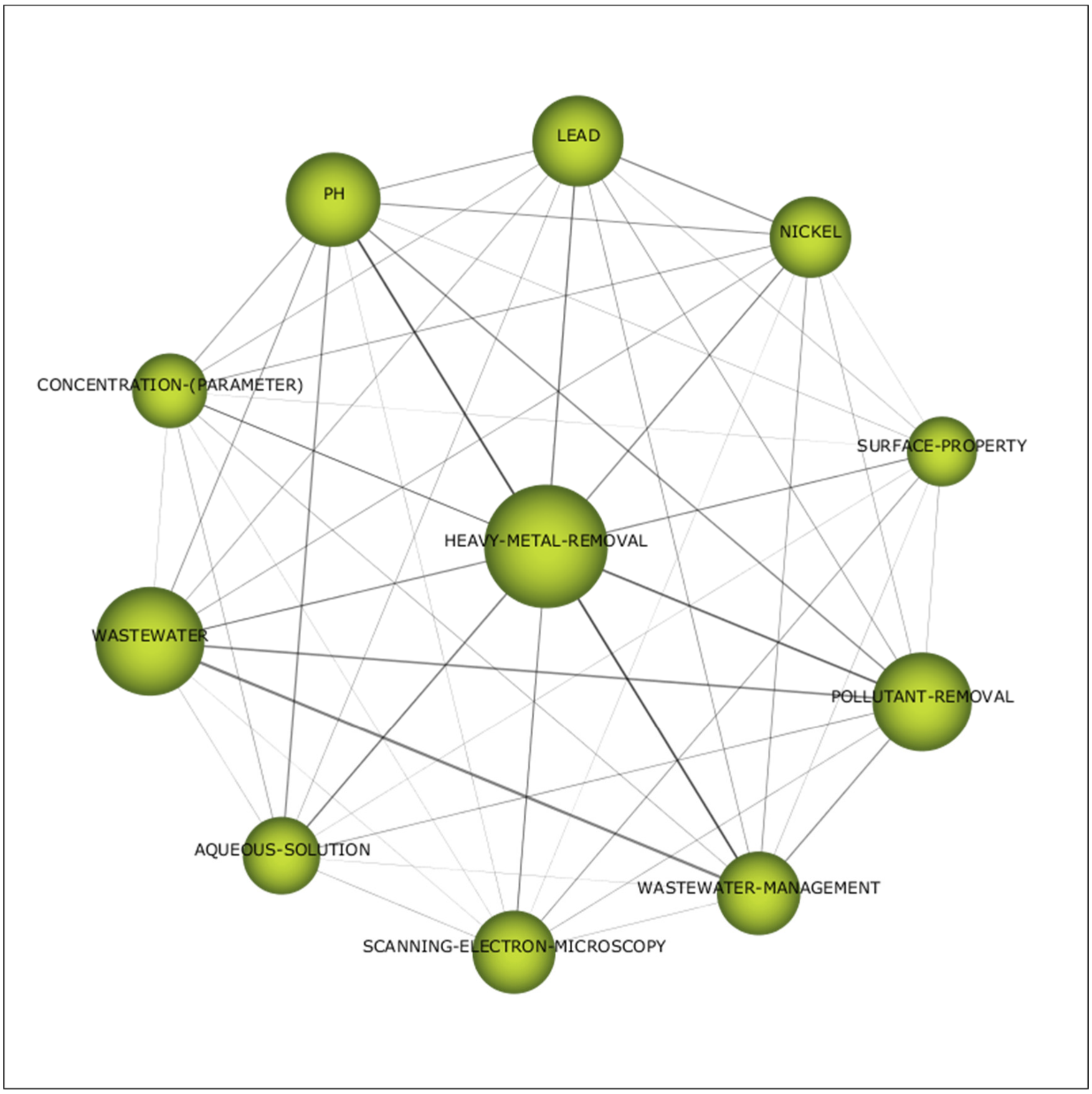

Figure S6. Evolution of the thematic network structure of the cluster Heavy Metal Removal (2011-2020).

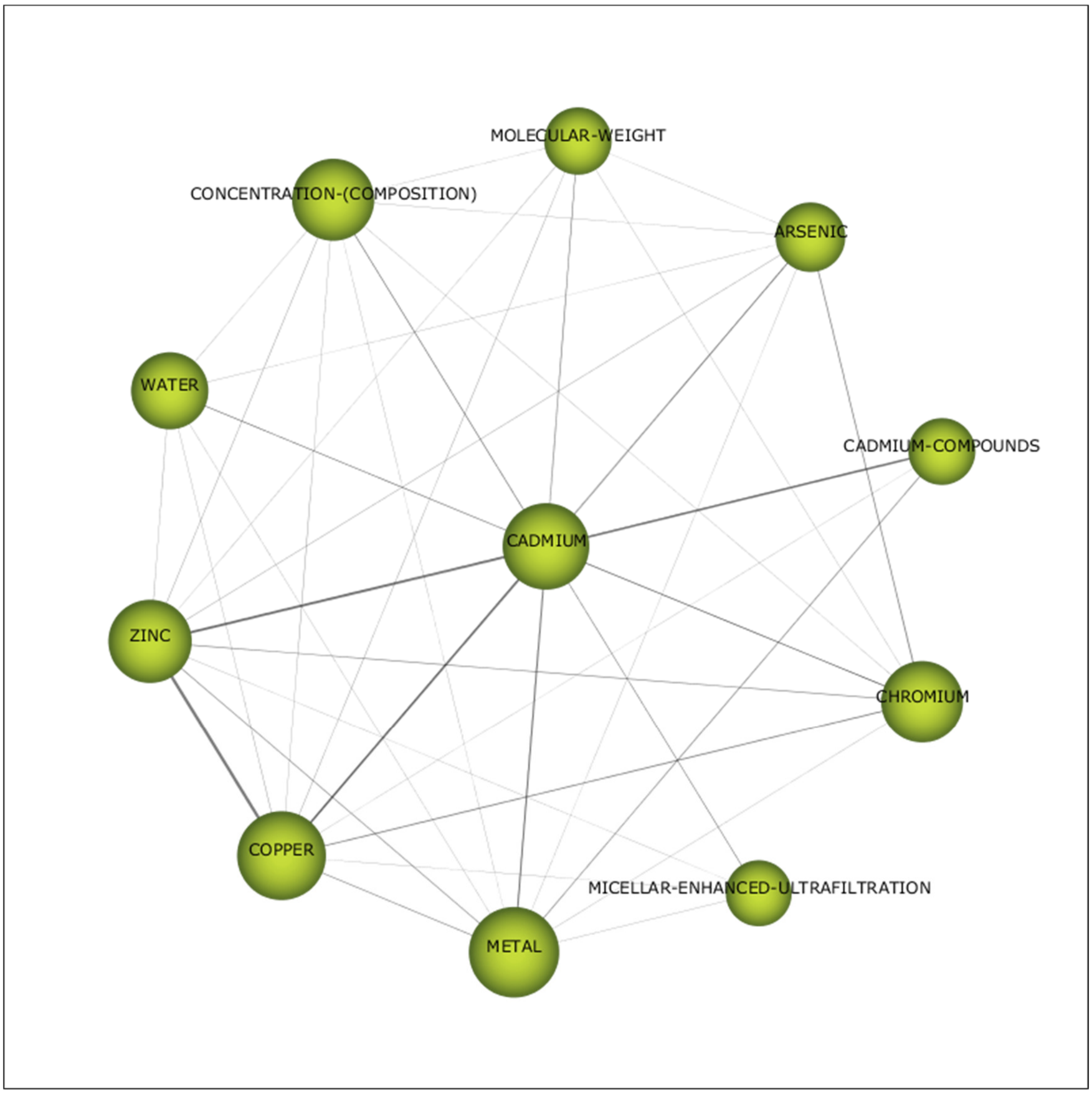

Figure S7. Evolution of the thematic network structure of the cluster Cadmium (2011-2020).

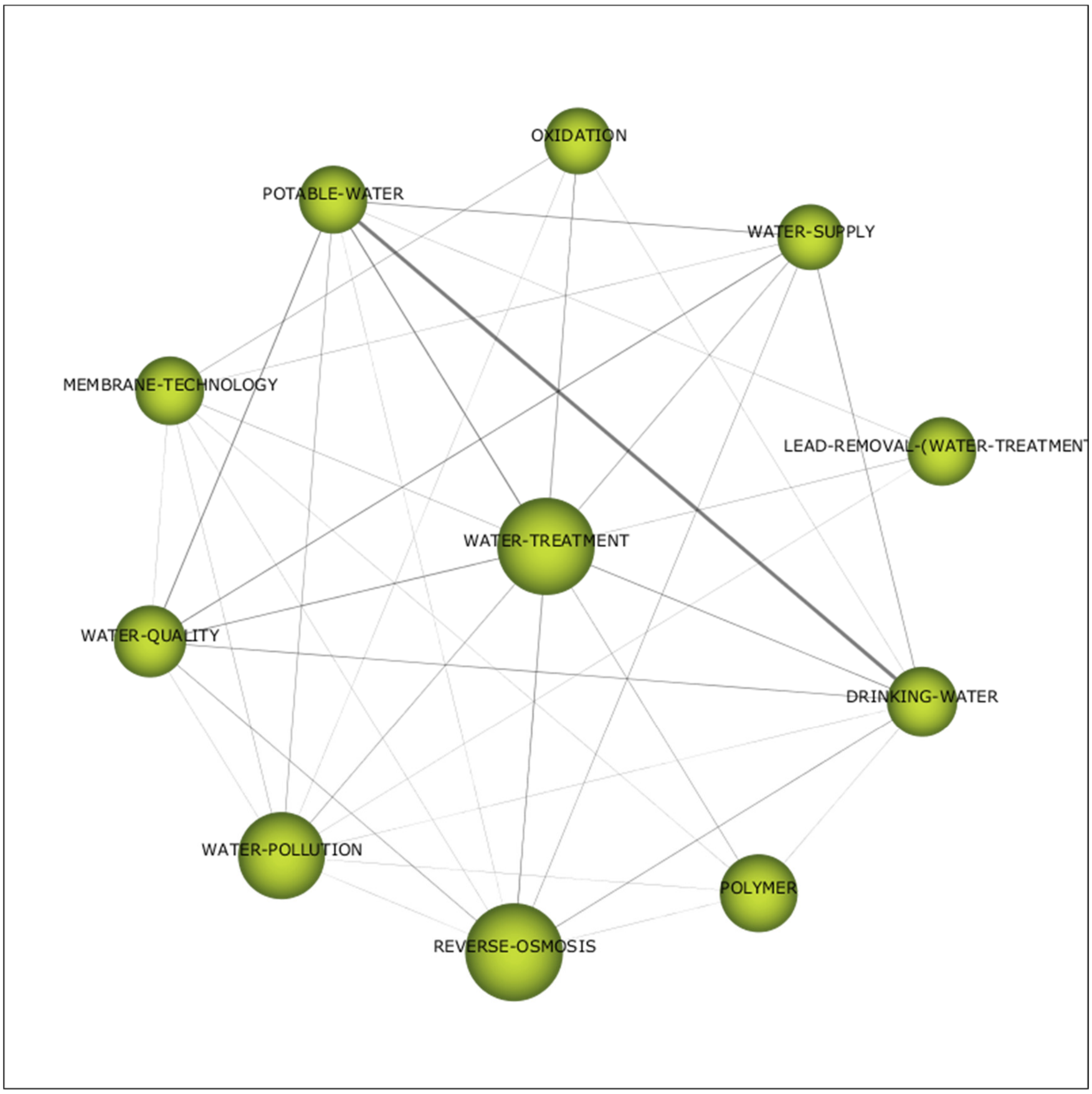

Figure S8. Evolution of the thematic network structure of the cluster Water Treatment (2011-2020).

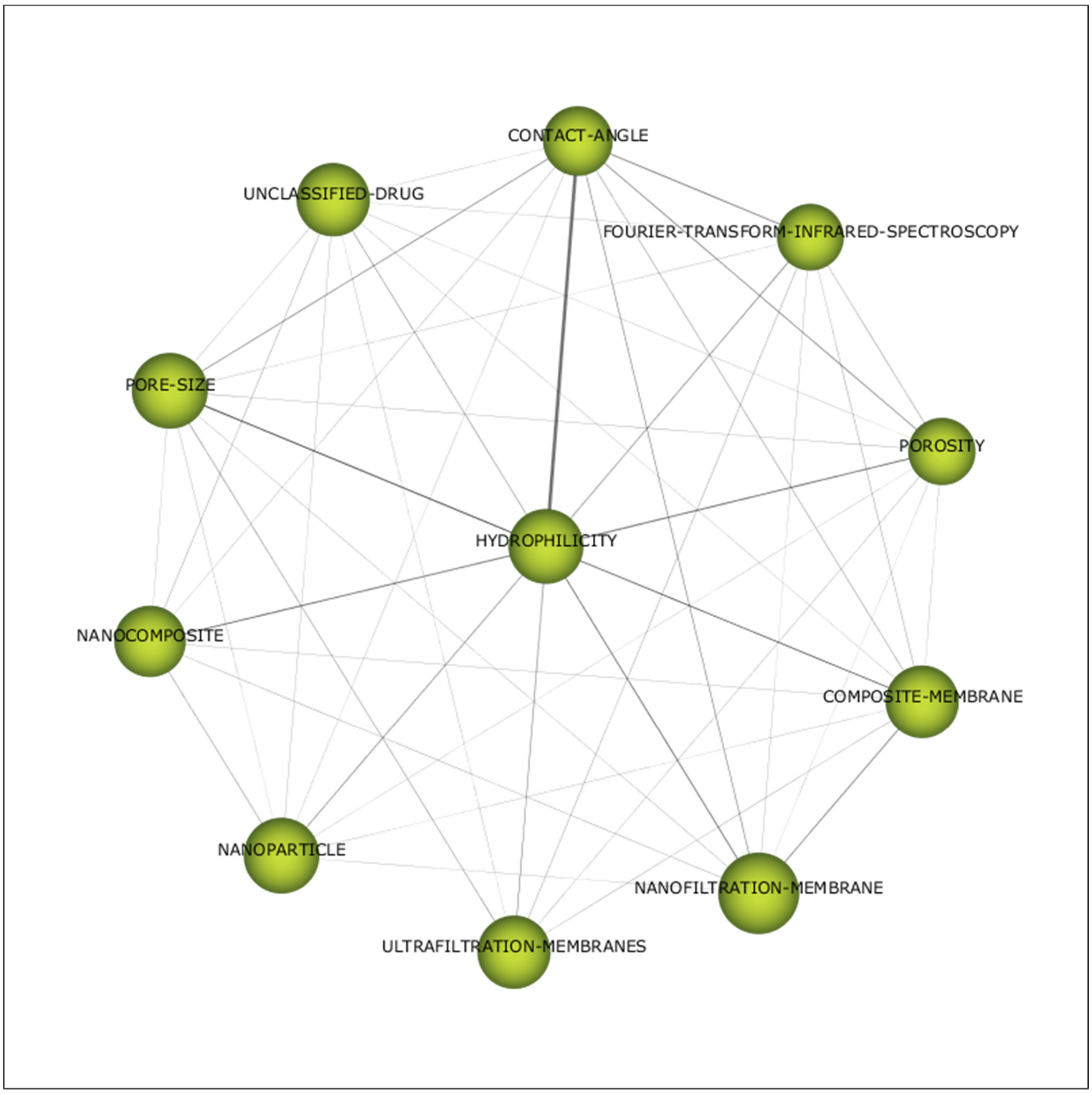

Figure S9. Evolution of the thematic network structure of the cluster Hydrophilicity (2011-2020).

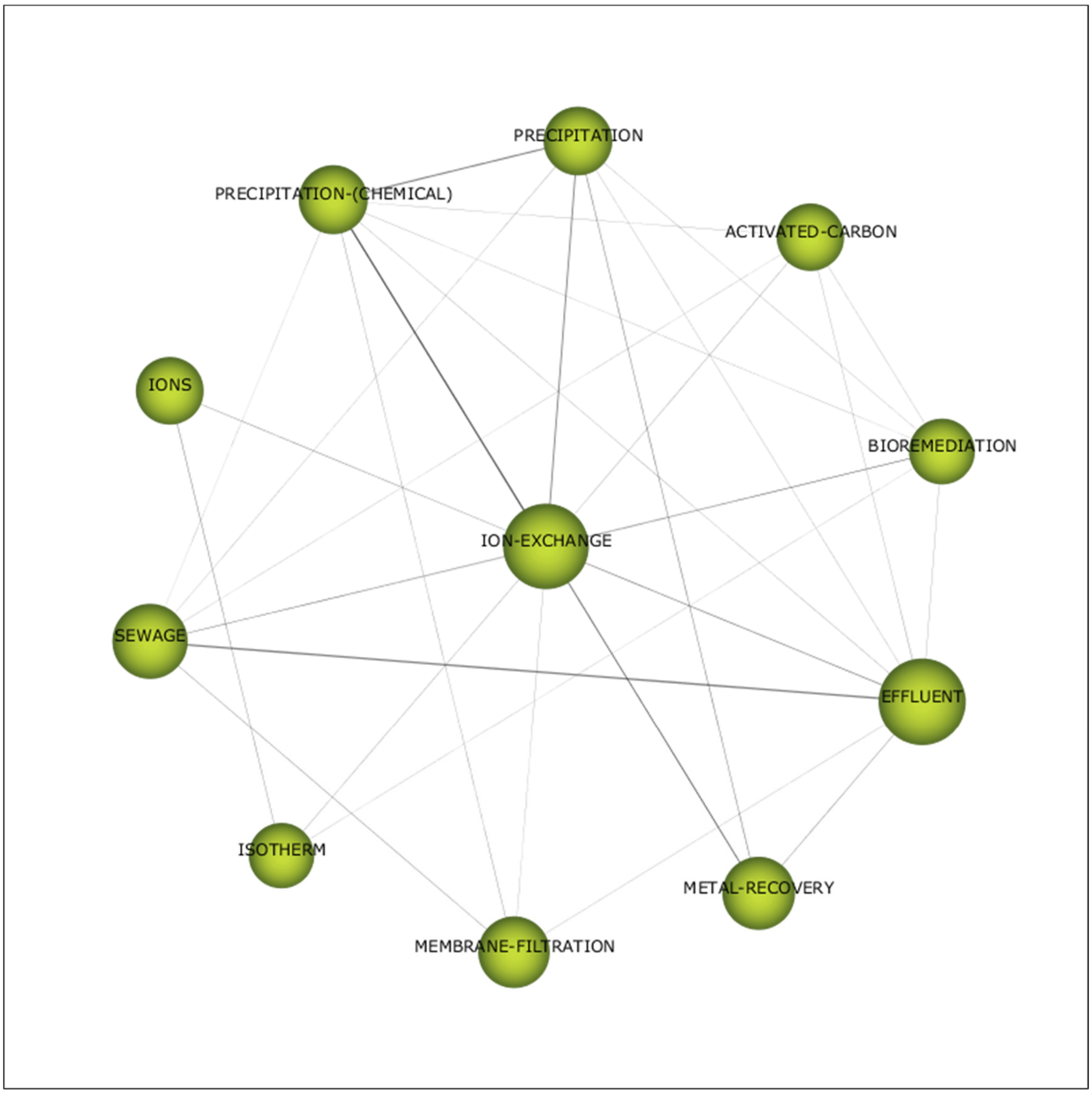

Figure S10. Evolution of the thematic network structure of the cluster Ion Exchange (2011-2020).

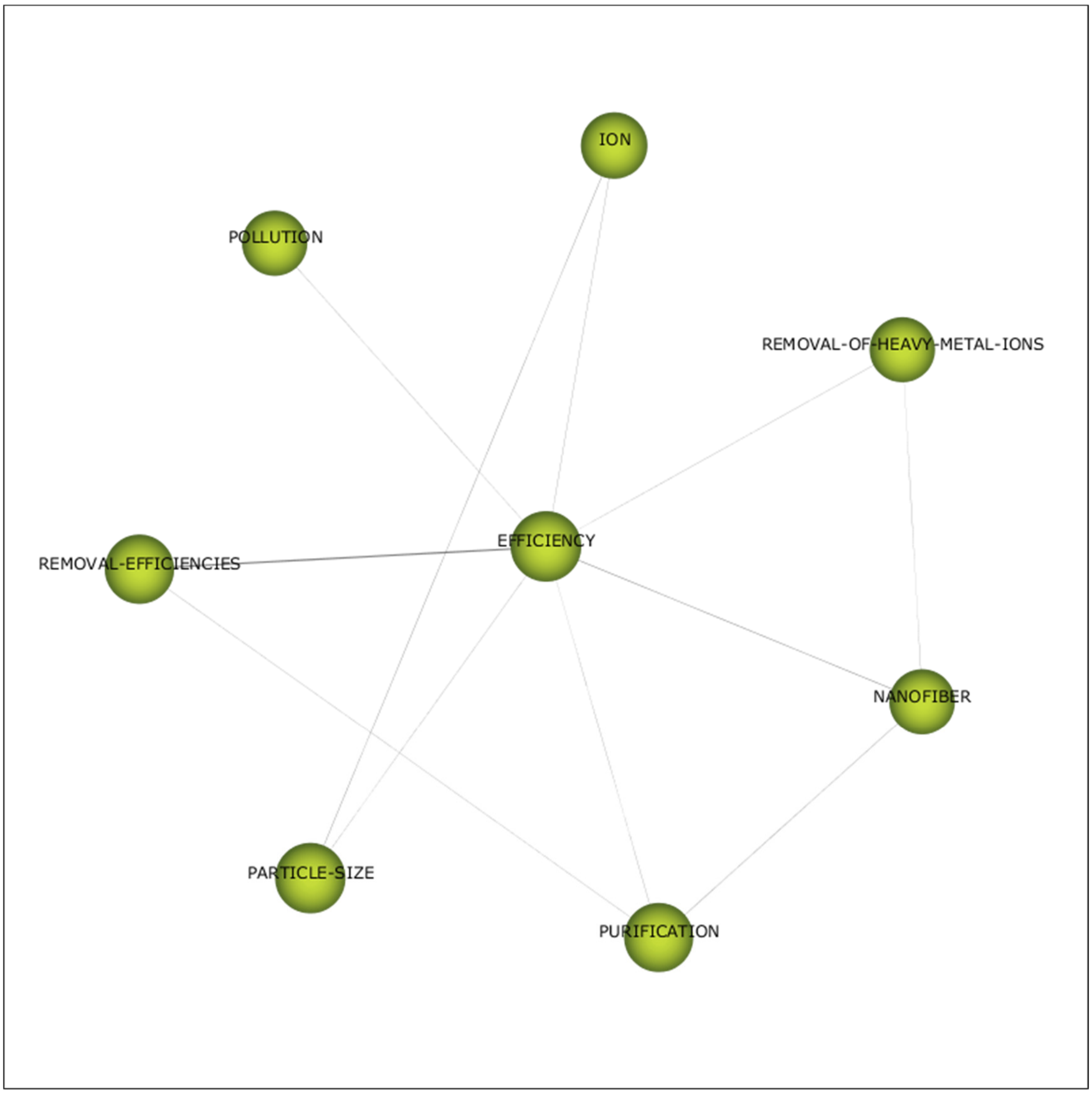

Figure S11. Evolution of the thematic network structure of the cluster Efficiency (2011-2020).

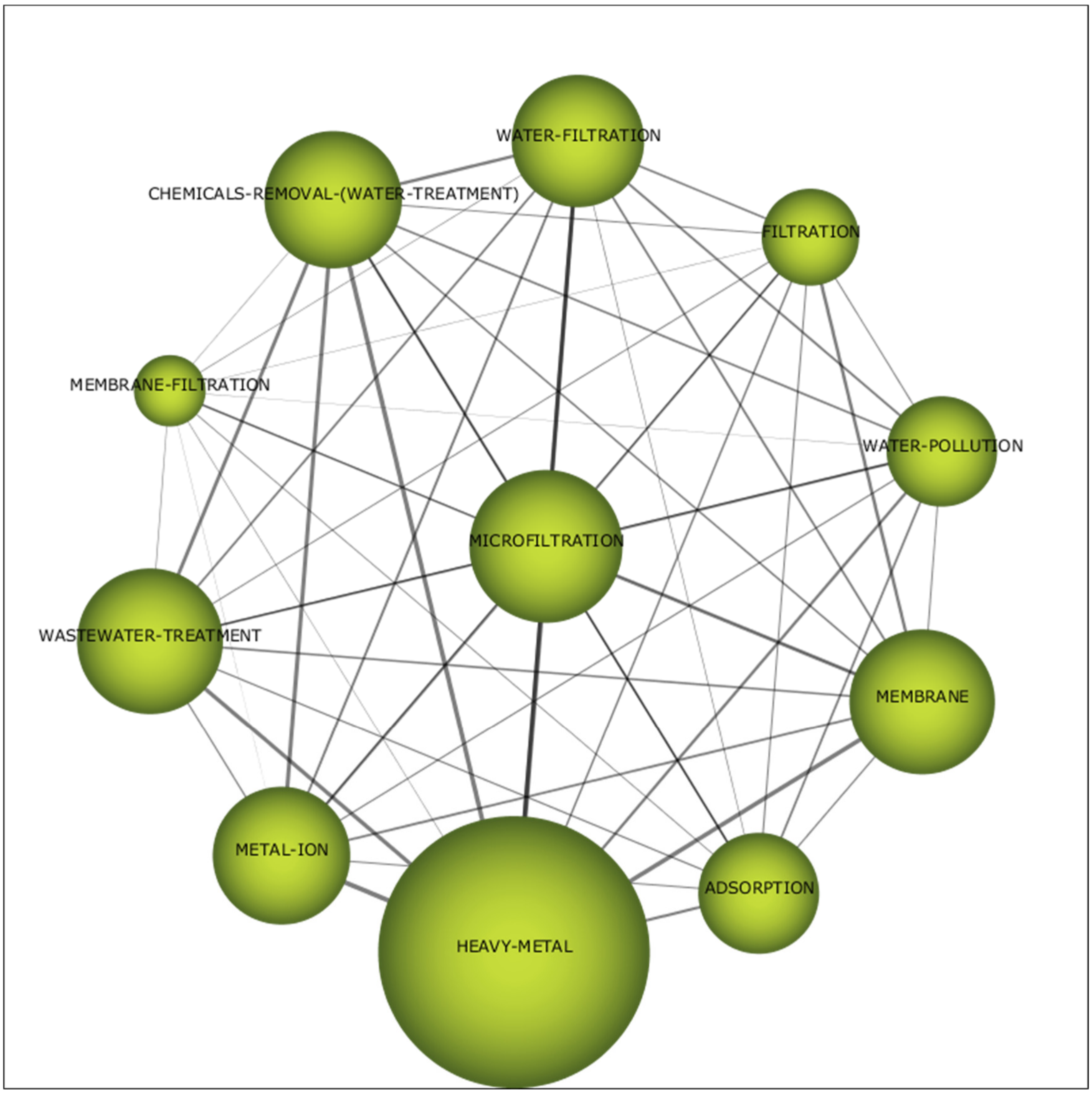

Figure S12. Evolution of the thematic network structure of the cluster Microfiltration (2021-2023).

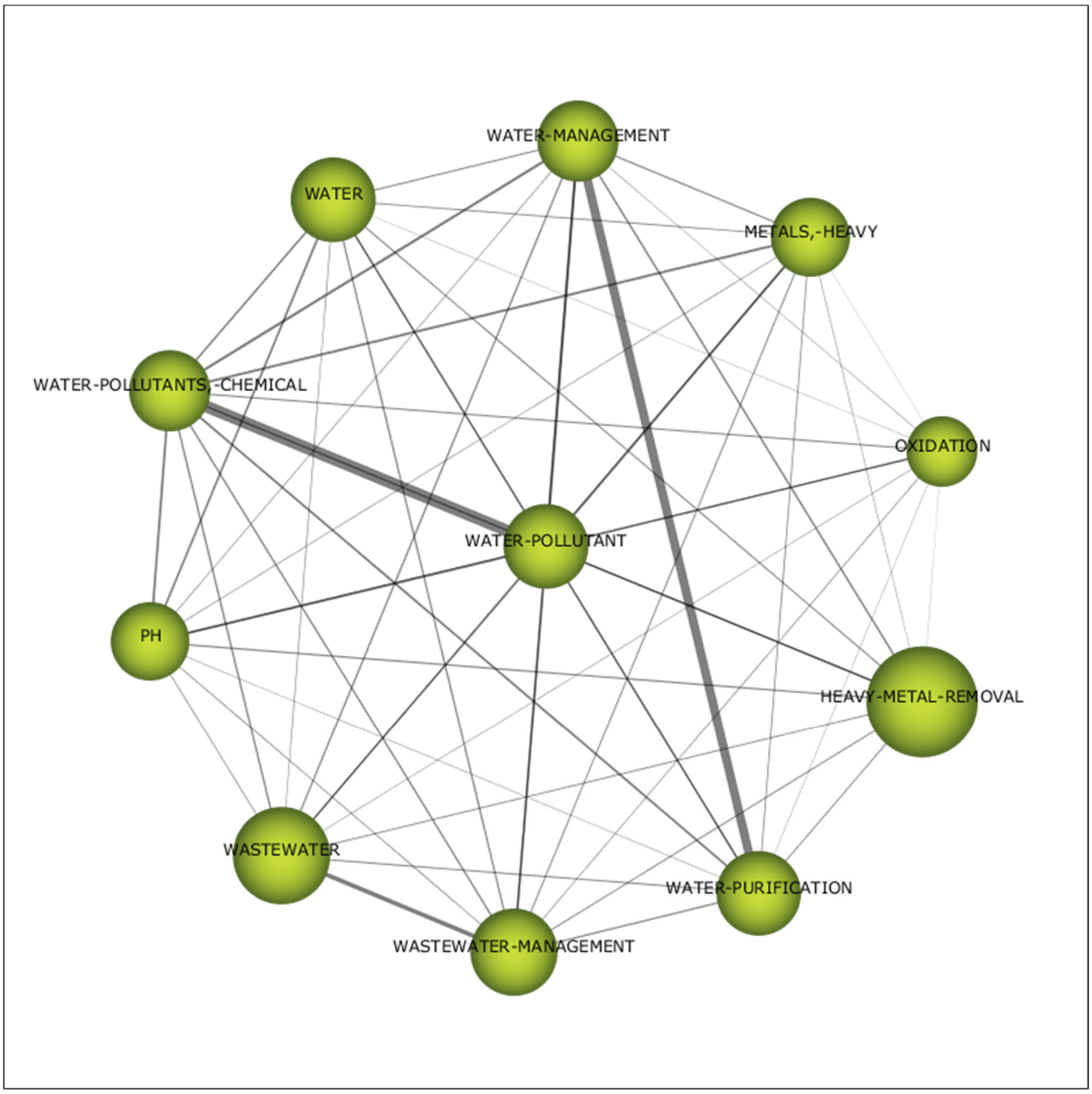

Figure S13. Evolution of the thematic network structure of the cluster Water Pollutant (2021-2023).

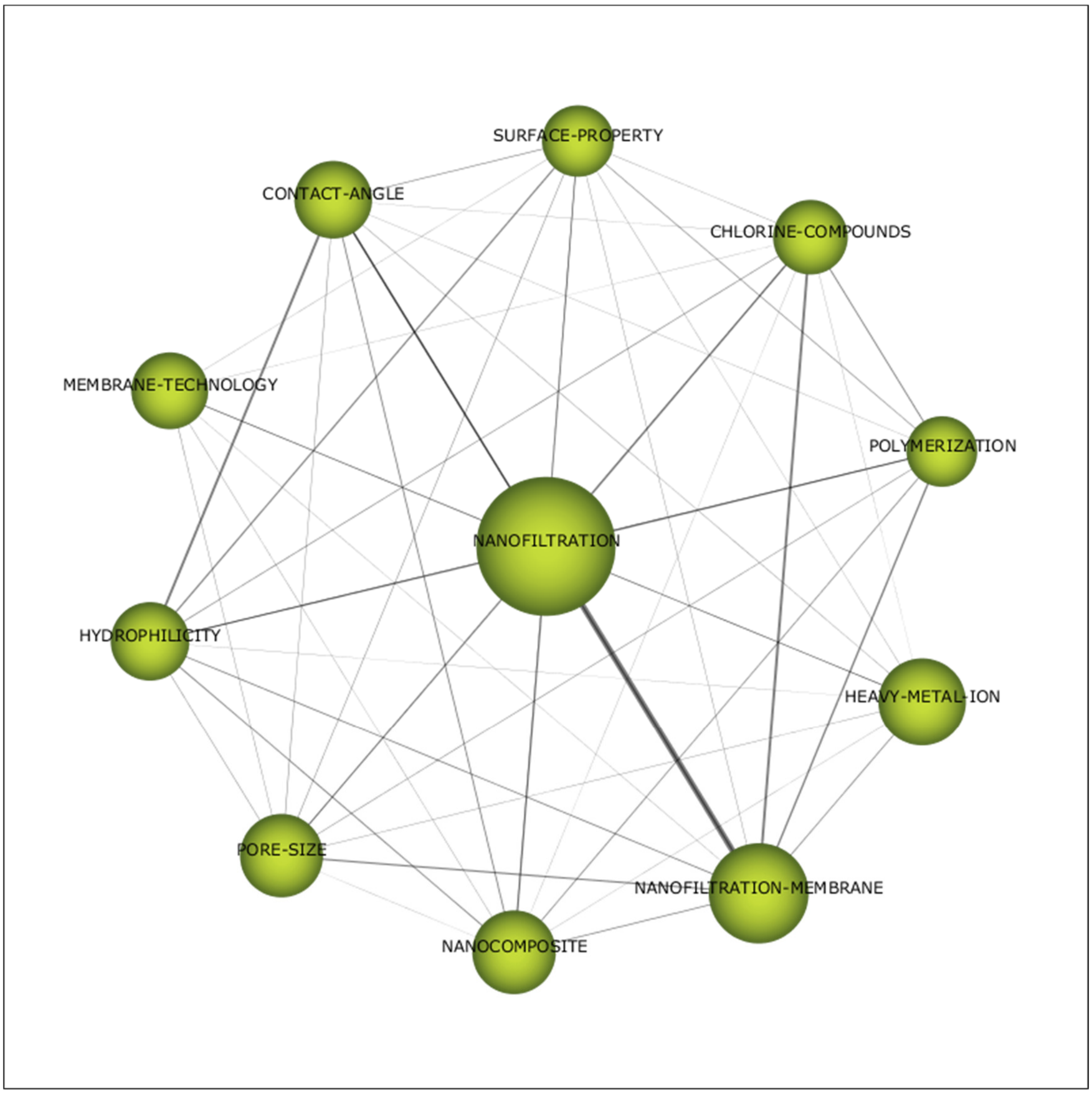

Figure S14. Evolution of the thematic network structure of the cluster Nanofiltration (2021-2023).

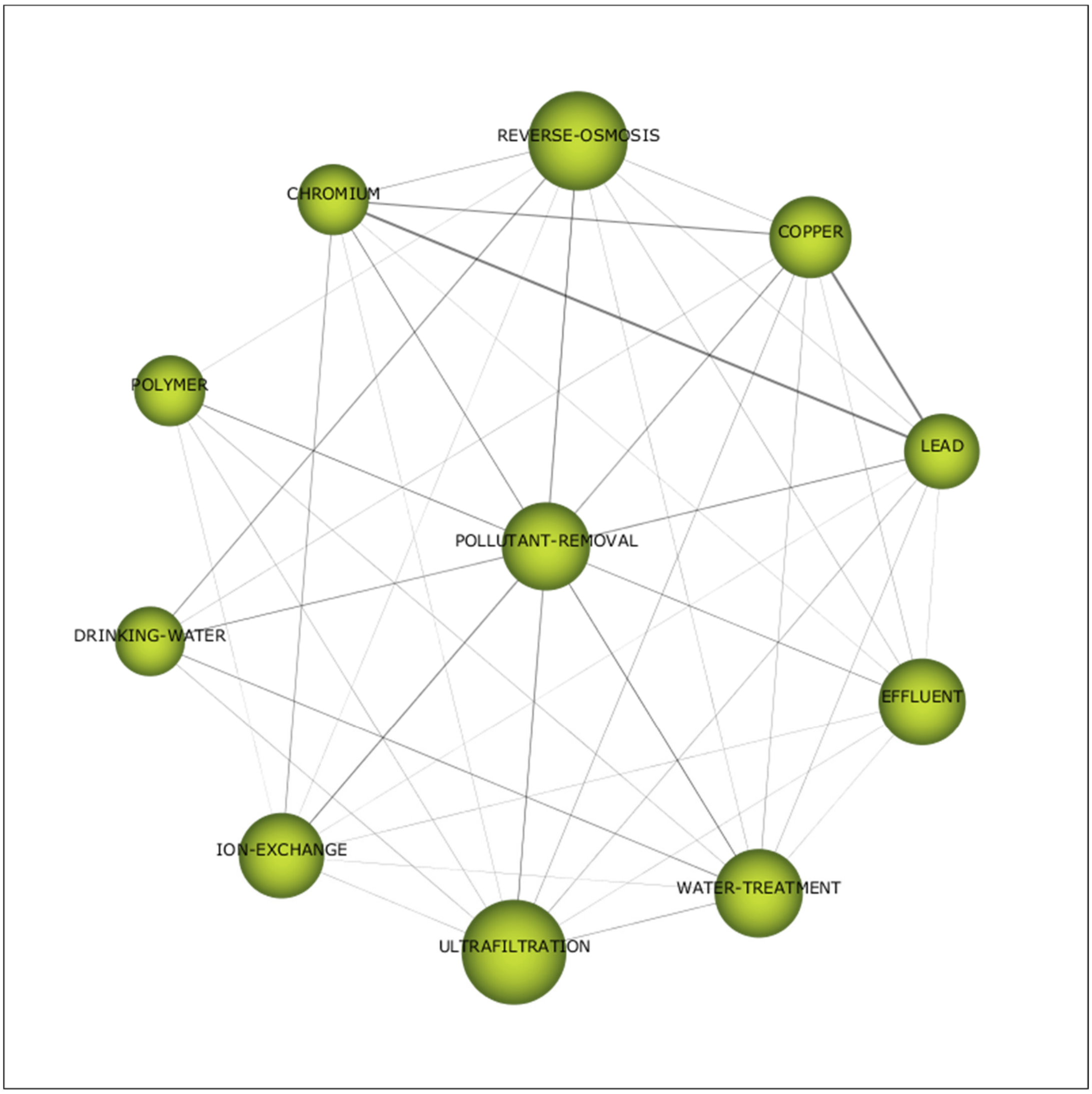

Figure S15. Evolution of the thematic network structure of the cluster Pollutant Removal (2021-2023).

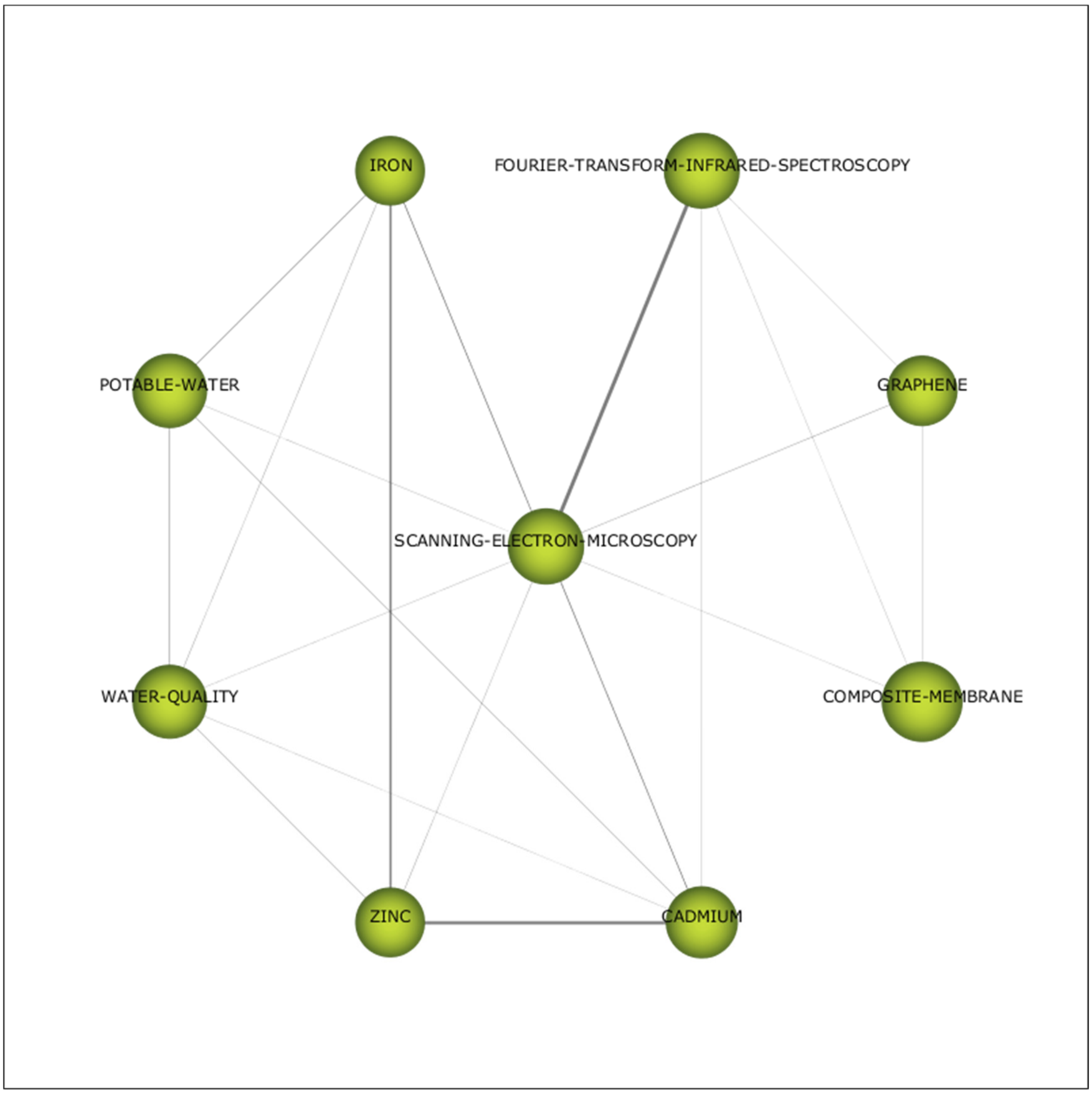

Figure S16. Evolution of the thematic network structure of the cluster Scanning Electron Microscopy (2021-2023).
